# Real roles of perylene diimides for improving photocatalytic activity†

**DOI:** 10.1039/d0ra03421e

**Published:** 2020-06-17

**Authors:** Fengxia Zhang, Wenjing Li, Tianyi Jiang, Xuemei Li, Yuanyuan Shao, Yongshan Ma, Junsen Wu

**Affiliations:** School of Municipal and Environmental Engineering, Shandong Jianzhu University Jinan 250101 Shandong China zhangfengxia19@sdjzu.edu.cn mlosh@sdjzu.edu.cn wujunsen@sdjzu.edu.cn

## Abstract

Three novel visible-light-driven composite photocatalysts: five-membered O-heterocyclic annulated perylene diimide doped TiO_2_ (PDI-1/TiO_2_), 1-phenol-*N*,*N*′-dicyclohexyl perylene-3,4,9,10-tetracarboxylic diimide doped TiO_2_ (PDI-2/TiO_2_), and *N*,*N*′-dicyclohexyl perylene diimide doped TiO_2_ (PDI-3/TiO_2_), were synthesized using a hydrothermal synthesis method. The effects of introducing PDIs with different structures into TiO_2_ were evaluated by assaying the photodegradation rate of Methylene Blue (MB). The photoactivities of the PDI-1/TiO_2_ and PDI-2/TiO_2_ catalysts were better than that of PDI-3/TiO_2_. This is because the large surface area of PDI-1 nanorods and PDI-2 nanobelts extended the 1D charge carrier channel, which facilitated electron transfer to the TiO_2_ surface and improved the photocatalytic activity of the composites. The PDI-1/TiO_2_ composite showed the highest photoactivity, and the activity remained at 86.4% after four reuse cycles. The extended π–π stacking of self-assembled PDI-1 and the strong interactions between self-assembled PDI-1 and TiO_2_ played significant roles in accelerating charge transfer and decreasing recombination of photogenerated electron–hole pairs. The steric hindrance of the phenoxy substituent at the bay position of PDI-2 prevented the PDI-2 nucleus from contacting TiO_2_ and weakened the interaction between PDI-2 and TiO_2_, which further resulted in the low photoactivity of PDI-2/TiO_2_. This work provides a practical way to improve the performances of traditional organic and inorganic composite photocatalysts.

## Abstract 2

Three novel visible-light-driven composite photocatalysts were synthesized by hydrothermal method. The effects of introducing PDIs with different structures into TiO_2_ were evaluated by assaying the photodegradation rate of methylene blue.

## Graphical abstract

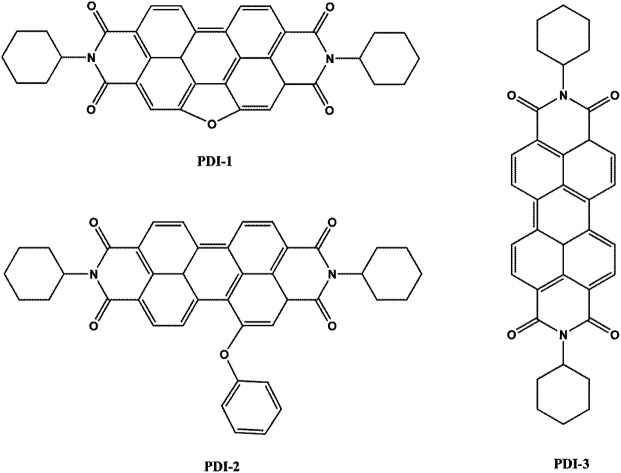

## 1. Introduction

Perylene tetracarboxylic diimide and its derivatives (PDIs) are considered to be good n-type organic semiconductor materials because of their excellent optical and thermal stabilities, excellent optical trapping abilities, and carrier transport properties.^1,2^ PDIs have high electron affinities and electron mobilities due to the strong π–π stacking between conjugated π-bonds. Therefore, they have been extensively used as fluorescent and near-infrared dyes, or as organic field effect transistors and photodiodes.^3,4^ The special fused-ring structures and strong intermolecular forces of PDIs make them poorly soluble, and they have high electron–hole recombination. These properties have limited their applications in photocatalysis.^5^

Modifications of the molecular structures of PDIs were usually achieved by introducing substituents into the imine nitrogen atoms or at bay locations.^6,7^ Substitutions at the imine position can increase the solubilities of PDIs in organic solvents and facilitate the assembly of PDI molecules into nanostructures. However, such substitutions can not affect the properties and electronic structures of PDIs because the nodes in both highest occupied molecular orbital (HOMO) and lowest unoccupied molecular orbital (LUMO) levels will limit the electronic interactions between substituents and perylene core.^8^ Substitutions at the bay position can also increase the solubilities of PDIs in organic solvents. In addition, such substitutions often result in distortion of the perylene core, which alters the photo-physical properties of PDIs.^9^ Moreover, phenol was often selected as a soluble group due to its flexibility and low steric resistance, and so it has a slight effect on intermolecular proximity and π–π stacking of adjacent PDIs. Sometimes phenoxy substituents can also facilitate the perylene molecules to self-assemble into ordered structure. The planar molecular geometries of PDIs with phenoxy substituents gave them high charge carrier mobilities, which could be a premise of efficient photoelectronic and organic electronic devices.^10^ As important structural motifs, fully fused bay-modifications of PDIs have received extensive attention and such fusions have been shown to provide excellent electronic and photoelectronic properties.^11^ The extension along the short molecular axis of perylene core caused a shift in the absorption spectrum and a variety of intermolecular interactions. These interactions are critical for achieving highly ordered supramolecular self-assembled structure which can enhance device performance.^12^ Introducing heteroatom into a π-conjugated system is also an easy way to construct intramolecular charge transfer compounds because the lone pair electrons of the heteroatom can be used as electron donors.^13^

Photocatalysis has received increasing attention due to its application in organic pollutants decomposing, water photo-splitting, and waste water treatment.^14^ Photocatalytic materials such as TiO_2_, ZnO, g-C_3_N_4_, metallophalocyanine (MPc), and their composites have been widely used.^15–19^ Among the reported photocatalytic materials, TiO_2_ is considered as an effective catalyst for the degradation of organic pollutants because it is non-toxic, inexpensive, and chemically stable. However, TiO_2_ has low visible light utilization efficiency and high electron–hole recombination efficiency, which limit its large-scale application.^20^ Surface adjustment strategies and modifications have been applied to TiO_2_ to increase its photocatalytic activity. For example, TiO_2_ doped with C, N, or metal particles exhibited improved catalytic activities. However, the response range of visible light did not increase significantly, and the charge recombination center can also be easily introduced for these modified TiO_2_.^21^ Therefore, they were still insufficient for practical applications.

Compared to traditional inorganic semiconductors, organic semiconductors have advantages like high flexibility, chemical structure diversity, and low cost. Introducing visible light active organic materials (such as PDIs) into TiO_2_ should be a possible approach to improve its solar photocatalytic activity.^22,23^ The main advantage of this method, similar to dye-sensitized nanocrystalline titanium dioxide solar cells, is that the interfacial area between the p- and n-type materials was greatly increased, and this change can improve the charging efficiency and the utilization rate of solar radiation. The low electron–hole recombination can also be improved by doping PDIs on TiO_2_. For example, Zang *et al.* deposited TiO_2_ layers on the 1D self-assembled nanofibers of PDIs *in situ*, which enhanced its hydrogen production activity.^24^ Nagarajan *et al.* synthesized *N*,*N*′-di(octadecyl)perylene-3,4,9,10-tetracarboxylic bisimide and TiO_2_ as a semiconductor material with π-conjugated structure and it could accelerate electron transfer during the degradation of Reactive Orange 4.^25^ Zhu *et al.* used TiO_2_ colloid and compound nanowires PDIs colloid under acidic conditions to improve the photocatalytic degradation activity towards organic pollutants.^26^ Cui *et al.* enhanced the photocatalytic degradation activity towards organic pollutants through self-assembly perylene tetracarboxylic acid diimide polymer nanostructures incorporating TiO_2_ nanoparticles.^27^ However, few literatures reported photocatalyst materials with bay area substituted PDIs, although such substituents can significantly change the electronic structures and photochemical properties of PDIs. It is because that this kind of PDIs was difficult to form well-defined nanostructures due to the distortion of π–π stacking.^28–30^ The researches on the mechanism of promoting photo-induced charge transfer by PDIs are still insufficient, and the separation efficiencies of electron and hole of the semiconductor catalysts are still low.

**Fig. 1:**
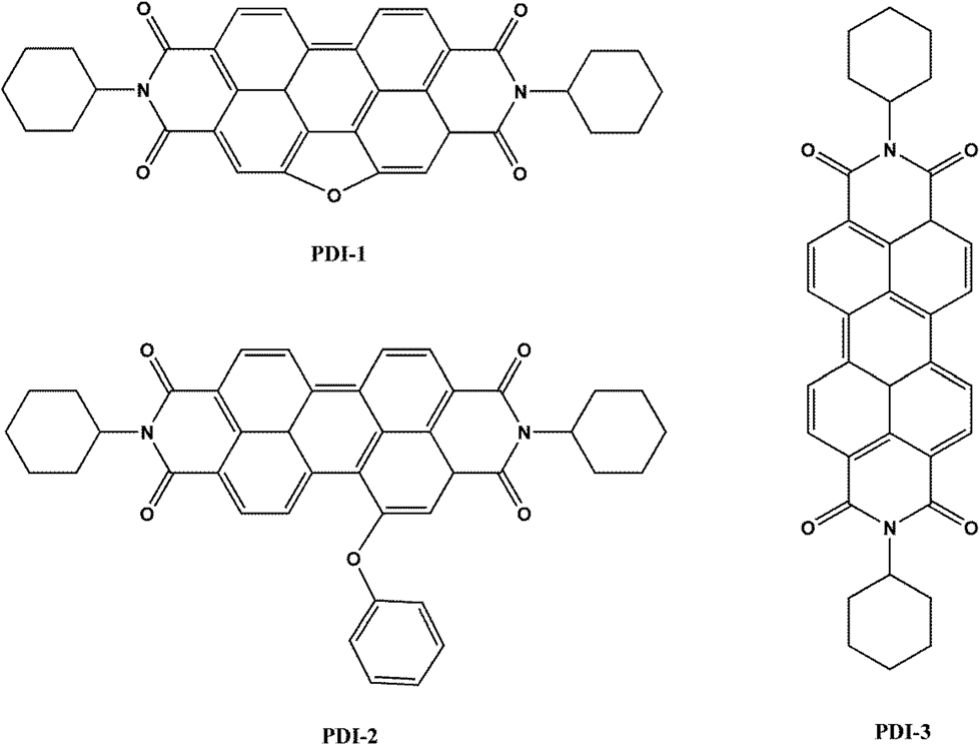
Chemical structures of PDI-1, PDI-2 and PDI-3 molecules.

Here, we designed and synthesized three novel visible-light-driven composite photocatalysts: five-membered O-heterocyclic annulated perylene diimide doped TiO_2_ (PDI-1/TiO_2_), 1-phenol-*N*,*N*′-dicyclohexyl perylene-3,4,9,10-tetracarboxylic diimide doped TiO_2_ (PDI-2/TiO_2_) and *N*,*N*′-dicyclohexyl perylene diimide doped TiO_2_ (PDI-3/TiO_2_) (Fig. 1). These composites were prepared by hydrothermal synthesis method. Optical properties and electronic structures of PDI-1, PDI-2, and PDI-3, nanostructure morphology, absorption, fluorescence, surface chemical properties, crystalline structure, and stability of PDI-1/TiO_2_, PDI-2/TiO_2_ and PDI-3/TiO_2_, and binding energy of PDI-1, PDI-2 and PDI-3 with TiO_2_ have been investigated. Also, the photocatalytic activities of PDI-1/TiO_2_, PDI-2/TiO_2_ and PDI-3/TiO_2_ have been tested by degrading Methylene Blue (MB) under visible light.

## 2. Experimental and characterization

### 2.1. Materials and measurements

All reagents and solvents were analytically pure, purchased from commercial sources and used without further purification. The synthesis of bay unilaterally extended PDI-1 and bay phenoxy mono-substituted PDI-2 was performed according to our literatures procedure with yields of *ca.* 30% and 90%, respectively.^31,32^ The synthetic routes of PDI-1 and PDI-2 (Fig. S1†) along with their characterization data are reported in the ESI Methods.†^1^H NMR and ^13^C NMR spectra were measured with a Bruker Advance 400 spectrometer in CDCl_3_ at room temperature. FT-IR spectrum was taken on a Bruker Tensor-27 spectrophotometer. Mass spectra were recorded in a Bruker Maxis UHR-TOF mass spectrometer. Absorption spectra and fluorescence measurements were performed at a Varian CARY-50 spectrophotometer and a Hitachi FL-4500 spectrometer. Cyclic voltammetry was recorded at a CHI760E electrochemical analyzer using three electrode cell units, Pt as counter electrode, glassy carbon working electrode and Ag/AgNO_3_ reference electrode. Tetrabutylammonium perchlorate (Bu_4_NClO_4_) was used as a supporting electrolyte, the scan rate employed was 100 mV s^−1^ and the current sensitivity was given as 0.01 μA. Scanning electron microscopy (SEM) images were obtained on a FEI NOVA NANOSEM 450 microscope. The specific surface area of the catalyst was analyzed using nitrogen adsorption at 77 K applying the BET (Brunauer–Emmett–Teller) method using a micrometrics ASAP 2020 V3.00 H. X-ray diffraction (XRD) measurements were performed using a Rigaku R-AXIS RAPID X-ray diffractometer. The ultraviolet-visible diffuse reflectance absorption spectra (DRS) were measured on a Shimadzu model UV-Vis diffuse reflectance spectrometer. X-ray photoelectron spectroscopy (XPS) was performed on a KRATOS model XSAM800 instrument. The quantum yields in the solid states were measured with the Hamamatsu spectrometer C11347 Quantaurus-QY. Fluorescence lifetimes were measured with the Hamamatsu spectrometer C11367. Both structure optimization and the property calculations were performed using Density Function Theory with B3LYP hybrid method and the standard 6-31G* basis set with the Gaussian 03 program package.^33–35^ The Mott–Schottky plots, photocurrent, and electrochemical impedance spectroscopy (EIS) were measured on a CHI760E electrochemical workstation using a three-electrode system. ITO deposited by photocatalyst was used as working electrodes, platinum wire as counter electrode, saturated calomel as reference electrode, and 0.1 M Na_2_SO_4_ aqueous solution was used as electrolyte. The photoelectric response of the sample was measured at 0.0 V, and the EIS was performed at an open circuit potential at a frequency of 0–10 000 Hz.

### 2.2. Preparation of PDIs loaded TiO_2_ photocatalyst

The 0.01 wt% PDI-1 loaded TiO_2_ (PDI-1/TiO_2_) composite was prepared by the hydrothermal synthesis method, taking tetrabutyl titanate as the starting material. 10 mL of tetrabutyl titanate was dissolved in 30 mL of anhydrous ethanol and to this solution 5 mL of water was added dropwise under vigorous stirring. Then 10 mg of PDI-1 dissolved in 5 mL of dichloromethane was added dropwise for 20 minutes under sonication. The resulting colloidal suspension was stirred for 12 h and then transfer to the hydrothermal kettle at 200 °C for 3 h. The composite obtained was filtered and dried in an air oven at 100 °C for 4 h. This catalyst contained 0.01 wt% of PDI-1. The 0.01 wt% PDI-2 loaded TiO_2_ (PDI-2/TiO_2_), 0.01 wt% PDI-3 loaded TiO_2_ (PDI-3/TiO_2_), 0.005 wt% of PDI-1 loaded TiO_2_ (0.005 wt% PDI-1/TiO_2_), 0.02 wt% of PDI-1 loaded TiO_2_ (0.02 wt% PDI-1/TiO_2_) and pure TiO_2_ were prepared using the same procedure.

### 2.3. Irradiation procedure

A photochemical reactor (PhchemIII, Beijing, China NBeT) was used for the degradation by visible light. The visible light source is obtained by a 500W xenon lamp (XE-JY500) with cutoff filter (>420 nm). It has a reaction chamber with a specially designed reflector made of highly polished aluminium and a built-in cooling fan. It also provides a magnetic stirrer and 50 mL reactive quartz tubes. The light exposure length is 230 mm. The photodegradation reactions were carried out in quartz tube reactor with a 50 mL 10 mg L^−1^ MB solution and 50 mg photocatalyst powders. The reaction temperature was held at 25 °C with magnetic stirring.

The suspension solutions were stirred for 30 min in dark prior to illumination in order to reach the adsorption–desorption equilibrium. At given time intervals, 2.0 mL of the sample were withdrawn and centrifuged to remove the photocatalysts. The changes in the concentration of MB were monitored by UV-visible spectrophotometer from its characteristic absorptions at 291 nm and 664 nm. The absorbance at 664 nm is used to monitor the decolourization of MB, while the absorbance at 291 nm represents the aromatic part of MB and its decrease indicates the degradation of the aromatic part of the dye.

## 3. Results and discussion

### 3.1. Photocatalytic activity

The photocatalytic activity of PDI-1/TiO_2_, PDI-2/TiO_2_, and PDI-3/TiO_2_ were tested by degrading Methylene Blue (MB: C.I. no. 52015, molecular formula: C_16_H_18_ClN_3_S, molecular weight: 319.85), which has been extensively used in dyeing industry. Its structure and UV-visible spectra are shown in Fig. S9.†

**Fig. 2:**
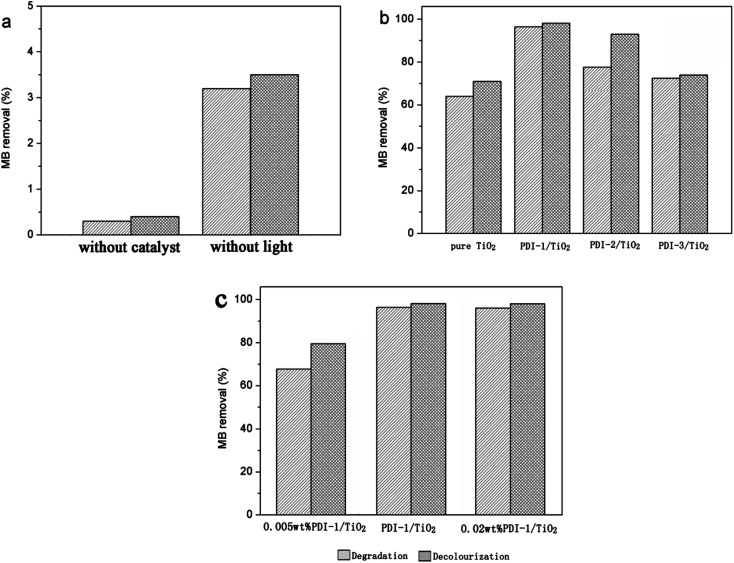
Photodegradation and decolourization of MB: (a) without catalyst or without light; (b) different photocatalysts; and (c) PDI-1/TiO_2_ composite photocatalysts with different PDI-1 mass ratio. [MB] = 10 mg L^−1^, pH = 7, catalyst suspended = 1 g L^−1^, irradiation time = 120 min.

Photocatalytic degradation and decolorization of MB with pure TiO_2_, PDI-1/TiO_2_, PDI-2/TiO_2_, and PDI-3/TiO_2_ under visible light are shown in Fig. 2. There was negligible degradation (about 0.3%) when the dye solution was irradiated without a catalyst. In the same experiment with PDI-1/TiO_2_ in the absence of light, the removal rate of MB was 3.5%, which may be due to the adsorption of dye on the catalyst (Fig. 2a). MB dye underwent 96.4% degradation and 98.1% decolorization in the presence of PDI-1/TiO_2_ under visible light irradiation for 120 min. These observations indicate that both light and photocatalyst were necessary to effectively degrade MB. The corresponding data was 78% and 93% for PDI-2/TiO_2_, and 73.9% and 72.4% for PDI-3/TiO_2_. Comparatively, only 64.1% degradation and 71% decolorization occurred with pure TiO_2_ as catalyst (Fig. 2b). Though TiO_2_ had no visible light absorption, it had been found to be solar active. This may be due to the higher adsorption of MB dye on the TiO_2_, which favored the presence of dye sensitization mechanism. These data show that the photocatalytic activity of the hybrid photo-catalyst PDI-1/TiO_2_ was obviously higher than that of other catalysts.

Fig. 2c showed the photocatalytic degradations of MB with 0.005 wt% or 0.02 wt% PDI-1/TiO_2_ under visible light irradiation for 120 min. Only 67.8% degradation and 79.5% decolorization of MB were observed with 0.005 wt% PDI-1/TiO_2_, whereas no significant change was observed with 0.02 wt% PDI-1/TiO_2_. The results showed that 0.01 wt% PDI-1/TiO_2_ had higher efficiency in MB degradation than other catalysts. For comparison, the pseudo-first order degradation rate constants of several common photocatalysts under the same conditions are also listed in ESI in Table S1.†

### 3.2. Characterization of catalyst

We were interested in why the 0.01 wt% PDI-1/TiO_2_ composite could gain the strongest oxidation ability and why the activity declined when PDI-1 was replaced by PDI-2 and PDI-3. The properties of these materials were determined by different PDIs molecular properties. To explain these results, the optical properties and electronic structures of the three PDIs were studied, and the morphologies of materials were observed to illuminate their internal structures.

**Fig. 3:**
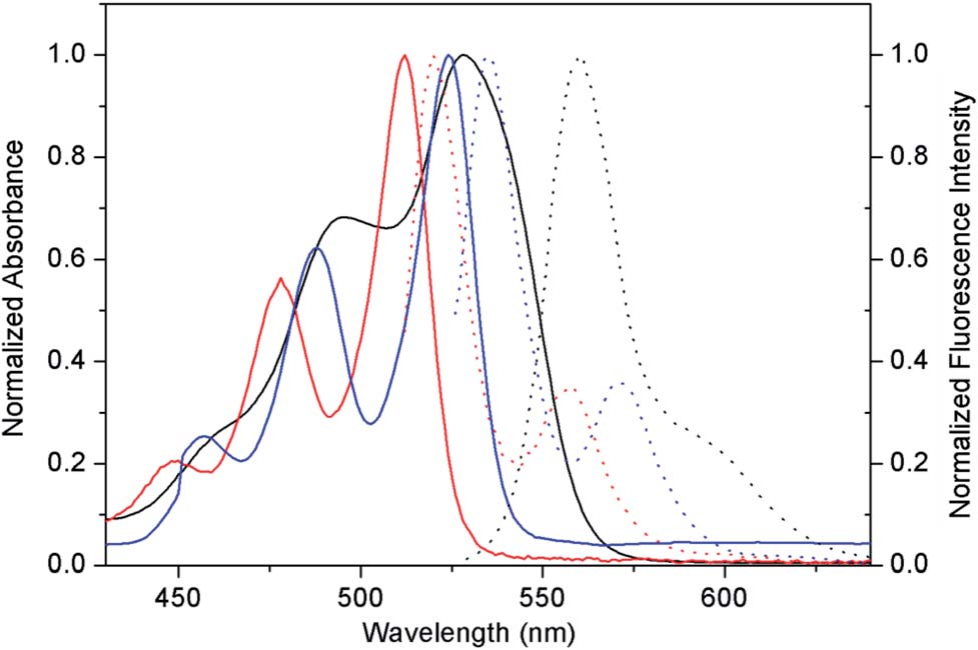
Optical properties of PDI-1, PDI-2, and PDI-3. Absorption spectra (solid lines) and corresponding fluorescence spectra (dot lines) of PDI-1 (red), PDI-2 (black), and PDI-3 (blue) in dichloromethane.

The optical properties of compounds PDI-1, PDI-2, and PDI-3 in dichloromethane were studied by UV-Vis and fluorescence spectra (Fig. 3). The spectra of PDI-3 showed two absorption bands (524 nm and 487 nm) and a shoulder peak at approximately 456 nm, which were consistent with the transition energy characteristics of 0–0, 0–1, and 0–2.^36^ The absorption peaks of PDI-1 appeared at 512 nm and 478 nm with a shoulder around 448 nm, which were blue shifted compared with PDI-3, reflecting the extension of aromatic nucleus on the short molecular axis.^37^ The maximum absorption peak of PDI-2 (558 nm) was red-shifted relative to PDI-1 and PDI-3. This change may be caused by the distortion of the PDI nucleus, or by the electronic coupling between the electron-rich substituent and the electron-deficient PDI nucleus.^38^ The fluorescence emission peaks appeared at 520 nm, 560 nm, and 535 nm for PDI-1, PDI-2, and PDI-3 with corresponding Stokes shifts of 8 nm, 32 nm, and 11 nm, respectively.

The absorption spectra of PDI-1, PDI-2, and PDI-3 in dichloromethane at various concentrations are shown in Fig. S10.† For a free monomer, the normal progression of its Franck–Condon factor was *A*^0–0^ > *A*^0–1^ > *A*^0–2^. However, as the monomers begin to aggregate, the 0–1 transition will increase.^31^ The 0–1 transition absorptions of PDI-2 (from 5.0 × 10^−6^ to 2.0 × 10^−5^ mol L^−1^) and PDI-3 (from 5.0 × 10^−6^ to 4.0 × 10^−5^ mol L^−1^) increased obviously (Fig. S11a and b†). However, the absorption of PDI-1 (from 5.0 × 10^−6^ to 2.0 × 10^−5^ mol L^−1^) did not show any increase (Fig. S11a†). These results indicated that aggregation occurred for PDI-2 and PDI-3 but didn't occur for PDI-1 at low concentration, and the solubility of PDI-1 was better than that of PDI-2 and PDI-3 in common organic solvent.

**Fig. 4:**
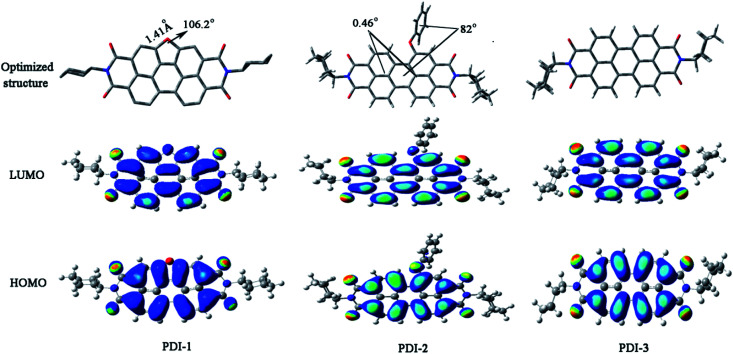
Optimized structures and computed frontier orbitals of PDI-1, PDI-2, and PDI-3 obtained by DFT calculations at the B3LYP/6-31G* level.

Optimized structures and computed frontier orbitals of PDI-1, PDI-2, and PDI-3 are shown in Fig. 4. The results show that PDI-1 has planar conformation. The formation of the O-heterocyclic ring induced the two naphthalene moieties to get far away in the site of open ring and induced the two naphthalene moieties to get close to each other in the site of O-heterocyclic ring. Planar conformation is favorable for the π–π stacking of self-assembled PDI-1 and covalent interactions between PDI-1 and TiO_2_. PDI-3 also had a planar conformation. But in PDI-2, the introduction of phenol substituent broke the original conformation. The approximate dihedral angle between phenol and perylene core was 82°, and the approximate dihedral angle between the two naphthalene subunits connected to the central benzene ring is 0.46°. The steric hindrance of the phenoxy substituent at bay position prevented the perylene nucleus from contacting TiO_2_ and so the interaction between PDI-2 and TiO_2_ was weak. HOMO and LUMO orbital of PDI-2 and PDI-3 are mainly distributed on the perylene ring. The phenol group contributes little to the molecular orbital of PDI-2. It is worth noting that the HOMO orbital of PDI-1 is centered at the perylene ring system, while the LUMO orbital is delocalized at the perylene nucleus and oxygen atom site. This is conductive to the electron delocalization from oxygen atom to TiO_2_. Moreover, due to the existence of electron-rich O-heterocyclic, PDI-1 is richer in electron in comparison with PDI-3. Thus, PDI-1 can act as a good hole-transporting layer.^39^ The cyclic voltammograms of PDI-1, PDI-2, and PDI-3 are illustrated in Fig. S12.†PDI-1 has two reduction peaks, indicating that it can accept at least two electrons.^40^ The HOMO/LUMO energy levels of PDI-1, PDI-2, and PDI-3 were estimated to be −6.26/−3.62, −6.52/−4.10, and −6.43/−3.89 eV, respectively. The increased HOMO and LUMO energy level of PDI-1 would improve its hole-transfer ability.^41^ The strong visible absorption, unique planar structure, good solubility, and higher HOMO/LUMO energy level of PDI-1 indicated that it has good application value in the field of photocatalysis.

**Fig. 5:**
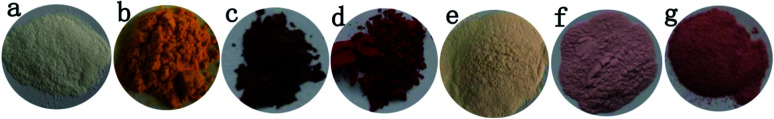
Photo images of TiO_2_ (a), PDI-1 (b), PDI-2 (c), PDI-3 (d), PDI-1/TiO_2_ (e), PDI-2/TiO_2_ (f) and PDI-3/TiO_2_ (g).

The color of pure TiO_2_, PDI-1, PDI-2, PDI-3, PDI-1/TiO_2_, PDI-2/TiO_2_, and PDI-3/TiO_2_ are shown in Fig. 5. The pure TiO_2_, PDI-1, PDI-2, and PDI-3 is white, yellow, dark red, and bright red, respectively (Fig. 5a, b, c and d), whereas PDI-1/TiO_2_, PDI-2/TiO_2_, and PDI-3/TiO_2_ is butter yellow, pink, and brownish red, respectively (Fig. 5e, f and g).

**Fig. 6:**
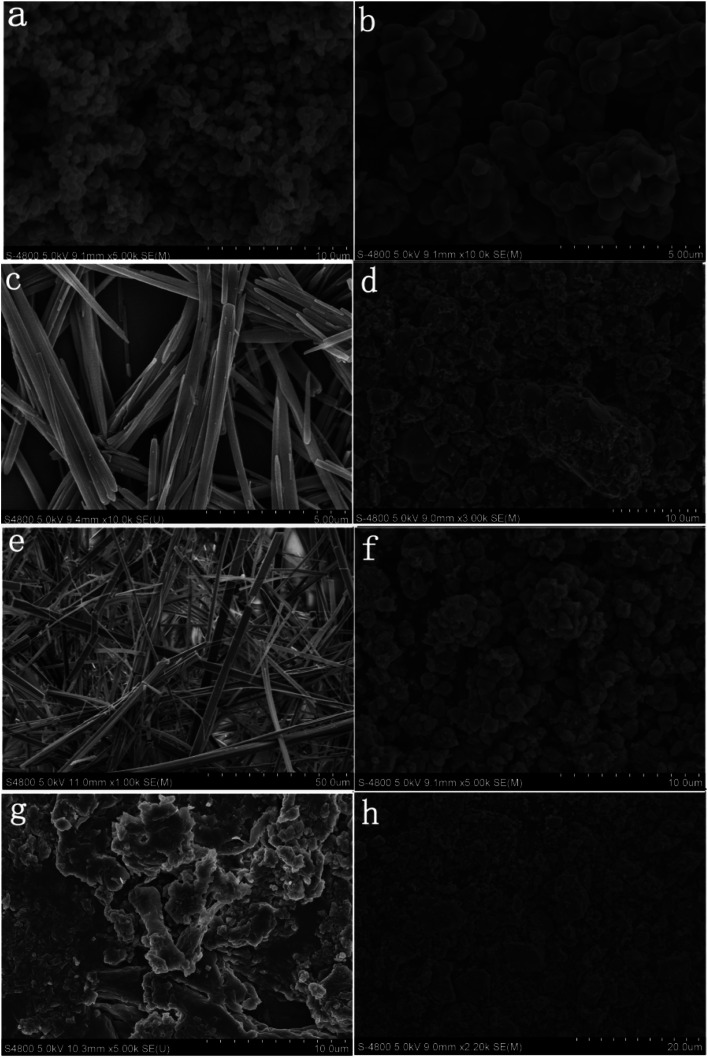
SEM images of particles of the TiO_2_ (a and b), PDI-1/TiO_2_ (d), PDI-2/TiO_2_ (f), and PDI-3/TiO_2_ (h) solid. SEM images of the PDI-1 (c), PDI-2 (e), and PDI-3 (g) in mixture solution (DCM : MeOH = 1 : 3).

Fig. 6 shows SEM images of TiO_2_, PDI-1, PDI-2, PDI-3, PDI-1/TiO_2_, PDI-2/TiO_2_, and PDI-3/TiO_2_. Fig. 6a and b shows that TiO_2_ form uniform spherical particles with diameter of about 500 nm. As can be seen from Fig. 6c, e and g, pure PDI-1, PDI-2, and PDI-3 in mixture solution (DCM : MeOH = 1 : 3) formed nanorods (the average width was 200 nm, and the length was in the range of a few tens of micrometers), nanobelts (the average width was 3 μm, and the length was in the range of 300 μm to 500 μm), and amorphous stack structure, respectively. The aggregation of the molecules seemed to be dominated by the PDI rings with substituent at bay position. Despite the different morphologies, both PDI-1 and PDI-2 nanostructures formed extended 1D charge carrier pathways (enabled by the π–π stacking) and had large surface areas, making them suitable for the study of comparative photocatalytic. 1D self-assembly of PDI-1 and PDI-2 occurred because of their decreased solubilities when a large amount of CH_3_OH was injected into their CH_2_Cl_2_ solutions. The insolubility could drive a strong intrinsic co-facial π–π stacking of the aromatic core (stacking along the long axis) in conjunction with the association between the side chains (aggregating along the short axis). Fig. 6d, f and h could illustrate many features. The sponge-like PDI-1, PDI-2, or PDI-3 clusters was uniformly embedded on TiO_2_ surface. Fig. 6h clearly shows that particles of PDI-3/TiO_2_ solid exhibited an uneven spherical structure. Fig. 6d and f demonstrates that PDI-1/TiO_2_ and PDI-2/TiO_2_ formed stacking spherical structures with a size of micrometers. Compared with PDI-2/TiO_2_ (1 μm), the spherical structure of PDI-1/TiO_2_ (2 μm) was thicker. It so happened that the more porous structure can provide more surface active sites.^42^ The surface areas of the composites were determined using the nitrogen gas adsorption method. The BET surface area of PDI-1/TiO_2_ (92.5 m^2^ g^−1^) was higher than that of PDI-2/TiO_2_ (87.4 m^2^ g^−1^) and PDI-3/TiO_2_ (82.3 m^2^ g^−1^). Increase of surface area could facilitate dye adsorption and increase photocatalytic activity to some extent.

**Fig. 7:**
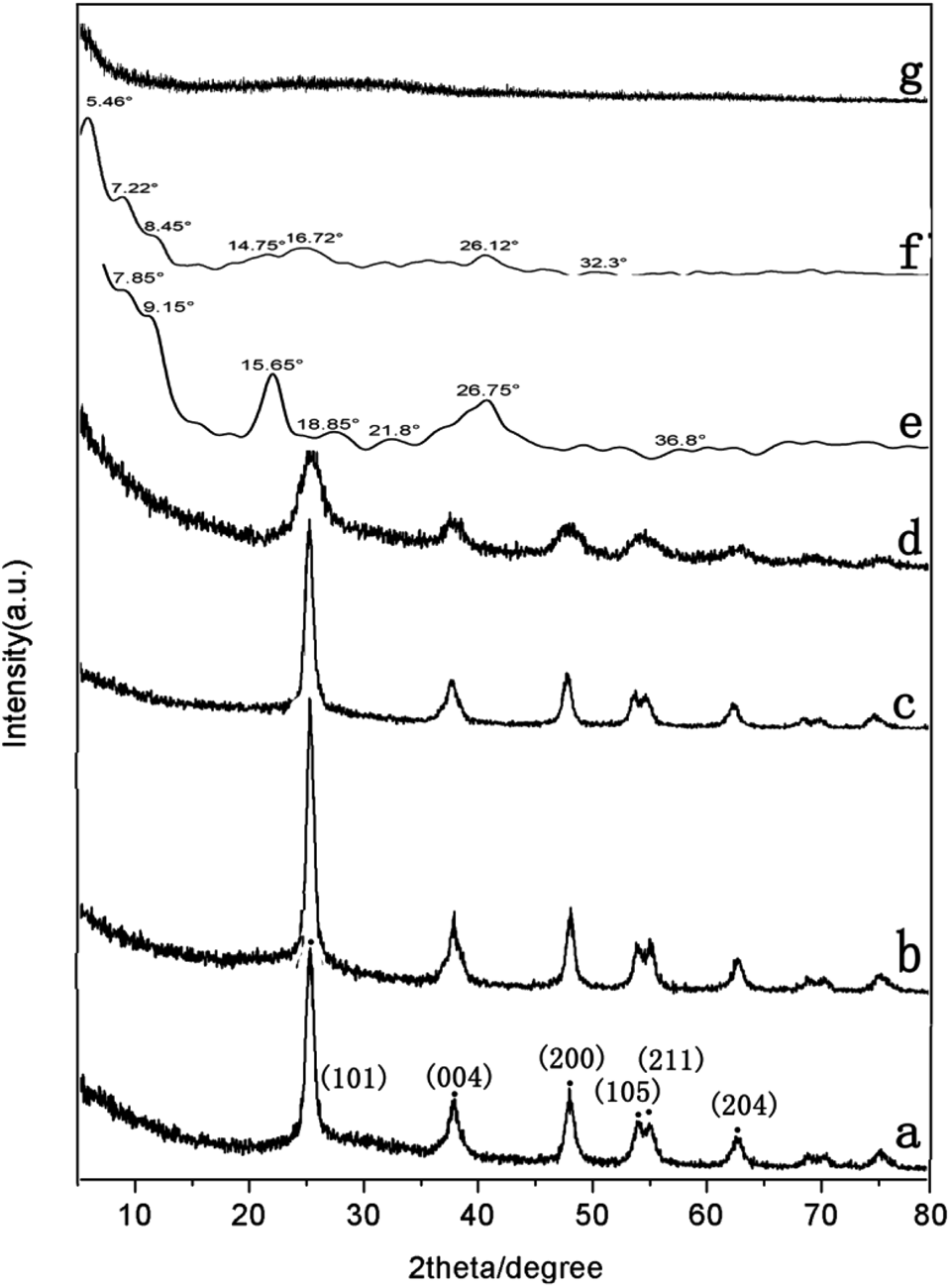
XRD patterns of (a) pure TiO_2_, (b) PDI-1/TiO_2_, (c) PDI-2/TiO_2_, (d) PDI-3/TiO_2_, (e) PDI-1, (f) PDI-2, and (g) PDI-3 composites.

**Fig. 8:**
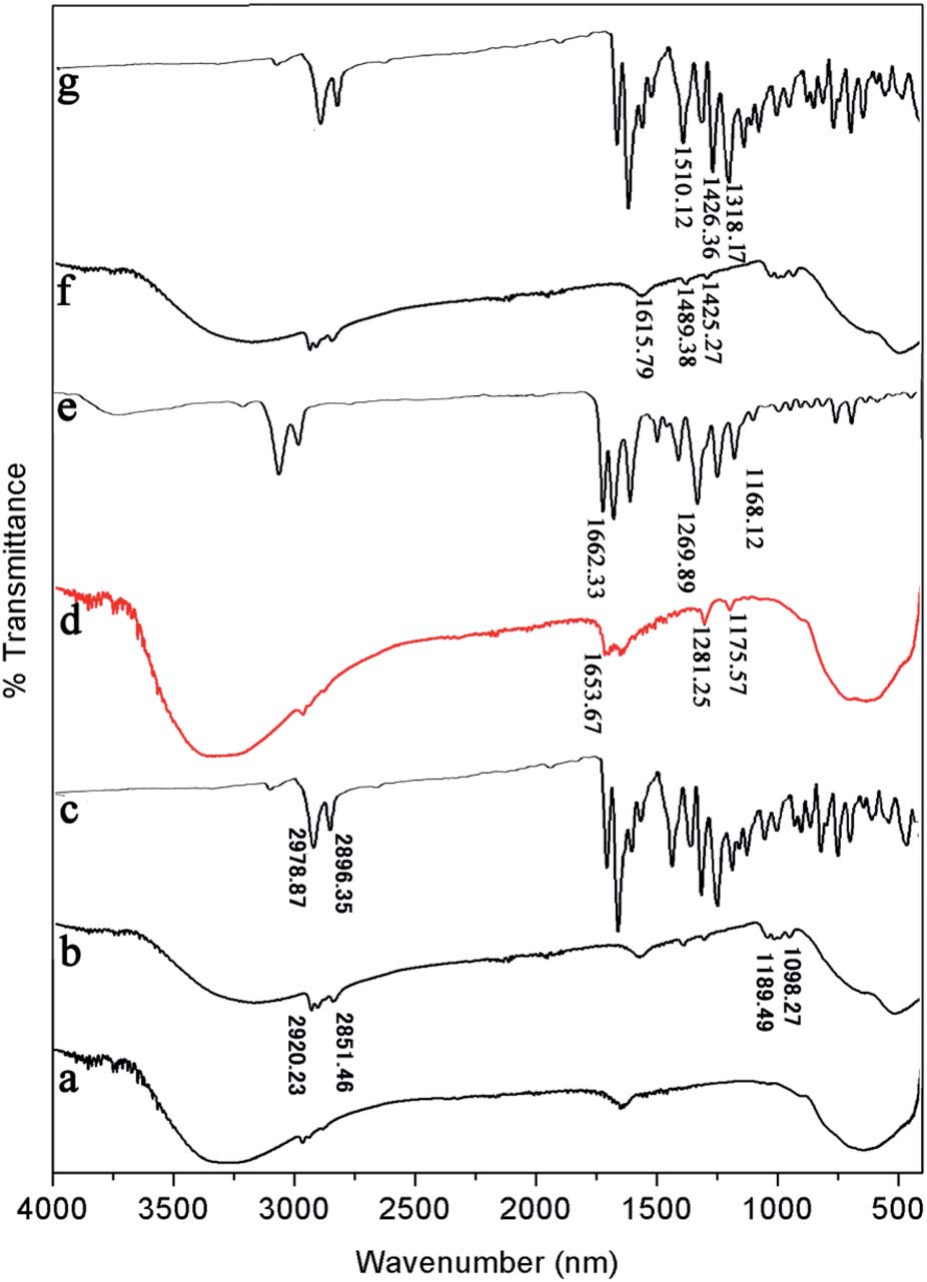
FT-IR spectra of (a) pure TiO_2_, (b) PDI-1/TiO_2_, (c) PDI-1, (d) PDI-2/TiO_2_, (e) PDI-2, (f) PDI-3/TiO_2_, and (g) PDI-3 composites.

XRD measurements were performed to determine the crystalline structure of pure TiO_2_, PDI-1, PDI-2, PDI-3, PDI-1/TiO_2_, PDI-2/TiO_2_, and PDI-3/TiO_2_ composites (Fig. 7). The crystal phase of TiO_2_ in the figure was consistent with the standard card (JCPDF no. 65-5714) (Fig. 7a). This phase could be characterized by the appearance of Bragg diffraction peaks at 2*θ* = 25.3°, 37.7°, 48.0°, 53.8°, 55.0°, and 62.6°, which were indexed to (101), (004), (200), (105), (211), and (204) planes for TiO_2_, respectively.^43^ Obviously, the PDI-1/TiO_2_ formed an ordered structure. Its crystallization property was enhanced in comparison with pure TiO_2_, which should be beneficial for its photocatalytic activity.^44^PDI-1/TiO_2_, PDI-2/TiO_2_, and PDI-3/TiO_2_ composites were doped with very little PDI-1, PDI-2, or PDI-3, and so there was no characteristic peak in the XRD of PDI-1/TiO_2_ (Fig. 7b), PDI-2/TiO_2_ (Fig. 7c), and PDI-3/TiO_2_ (Fig. 8d). In the X-ray diffraction pattern of PDI-1, the peak at 2*θ* = 26.75° (*d* spacing 3.3 Å) can be attributed to the π–π stacking of the adjacent perylene because the distances of π–π stacking between the perylene cores were approximately 3.5 Å.^45^ The intensity of this diffraction peak increased obviously for PDI-1. This result suggests that the π–π stacking interaction of PDI-1 units was powerful, and it was probably because of the unique planar structure of PDI-1 extended the π–π interactions. However, the intensity of this diffraction peak decreased obviously for PDI-2, suggesting that the π–π stacking interaction of PDI-2 units was weak. This is because the phenol substituent weakened the face-to-face arrangement of PDI units. In addition, XRD of PDI-1 showed that the first diffraction peak was 9.15°, the second diffraction peak was 18.85°, and the fourth diffraction peak was 36.8° (Fig. 7e). The multi-order reflections indicate that the self-assembled PDI-1 had well-ordered layered microstructures.^46^

FT-IR was also used to study the surface chemical properties of the composites (Fig. 8). The Ti–O–Ti characteristic absorption peak of TiO_2_ was located below 1000 cm^−1^, and the peak at 3200–3400 cm^−1^ was O–H peak, which was due to a small amount of water on the catalyst surface. The peaks in the range of 1000–1650 cm^−1^ corresponded to the skeleton vibration of PDI-1 benzene ring. The bending vibration peaks of C–H were at 2851 and 2920 cm^−1^.^47^ Compared with the spectrum of pure TiO_2_, three new peaks appeared at 1653.67, 1281.25, and 1175.57 cm^−1^ for PDI-2/TiO_2_ (and 1615.79, 1489.38, and 1425.27 cm^−1^ for PDI-3/TiO_2_). These peaks are characteristic peaks of PDI-2 (PDI-3). Compared with pure PDI-1, PDI-2, or PDI-3, the peaks of PDI-1/TiO_2_, PDI-2/TiO_2_, or PDI-3/TiO_2_ showed apparent shift, which were caused by the force between TiO_2_ and self-assembled PDI-1, PDI-2, or PDI-3. These peaks indicated the presence of PDI-1, PDI-2, or PDI-3 in the catalyst composites.

**Fig. 9:**
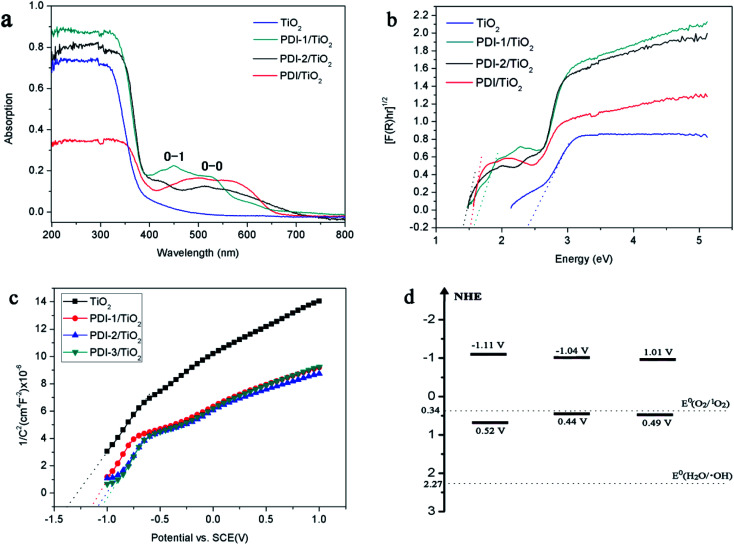
(a) DRS spectra, (b) (*F*(*R*)*hν*)^1/2^*versus hν*, (c) Mott–Schottky plots, and (d) diagram representing experimentally evaluated VB and CB levels of samples.

The absorptions of the catalysts were characterized by UV-Vis diffuse reflectance spectra (DRS) (Fig. 9a). UV/vis spectroscopy of the PDI-1/TiO_2_ revealed monomeric-dye absorption characteristics of PDI-1. It has absorption peaks identical to a progression of π–π transitions of perylene ring, designated as 0–0 and 0–1. For free monomers, the normal progression of Franck–Condon factor was *A*^0–0^ > *A*^0–1^. However, as monomers began to aggregate, the 0–1 transition increased.^48^ The 0–1 transition absorption of each of the three composites increased compared to its corresponding PDI monomer. These results suggest that aggregation occurred for PDI-1, PDI-2, or PDI-3 in corresponding catalysts. The band gaps (*E*_g_, eV) of the composites, by assuming an indirect transition between valence and conduction bands, were calculated using a Tauc equation and Kubelka–Munk function:^49^

1 [*F*(*R*)*hν*]^0.5^ = *A*(*hν* − *E*_g_)

2 *F*(*R*) = (1 − *R*)^2^/2*R*

where *F*(*R*) is proportional to the absorption constant, *R* is the calibrated reflection of samples with BaSO_4_ reflection, *h* is the Planck constant, *ν* is the frequency, *A* is constant, and *E*_g_ is the band gap energy.

The [*F*(*R*)*hν*]^0.5^*versus* the *hν* is shown in Fig. 9b. *E*_g_ value of the samples was obtained by extrapolating the linear part of the plot relating [*F*(*R*)*hν*]^0.5^ and *hν* to [*F*(*R*)*hν*]^0.5^ = 0. The band gaps are found to be 2.39, 1.59, 1.48, and 1.52 eV for TiO_2_, PDI-1/TiO_2_, PDI-2/TiO_2_, and PDI-3/TiO_2_, respectively. The increase in UV-visible light absorptions and decrease in band gap energies enhanced the photodegradation efficiency of the catalysts towards MB.

The bottom of conduction band (CB) of TiO_2_, PDI-1/TiO_2_, PDI-2/TiO_2_, and PDI-3/TiO_2_ can be estimated by the Mott–Schottky plots. As shown in Fig. 9c, the flat band potential (*E*_fb_) of TiO_2_, PDI-1/TiO_2_, PDI-2/TiO_2_, and PDI-3/TiO_2_ were −1.37 V, −1.11 V, −1.08 V, and −1.07 V *vs.* SCE (pH = 7), respectively. For n-type semiconductors, the CB was 0.2 V higher than that of Fermi level. Therefore, the CB of TiO_2_, PDI-1/TiO_2_, PDI-2/TiO_2_, and PDI-3/TiO_2_ under normal hydrogen electrode (NHE, pH = 7) can be calculated using the following equation:*E*_CB_ (NHE, pH = 7) = *E*_fb_ (SCE, pH = 7) + 0.24 − 0.2

The results showed that the CB position of TiO_2_, PDI-1/TiO_2_, PDI-2/TiO_2_, and PDI-3/TiO_2_ were about −1.33 V, −1.07 V, −1.04 V, and −1.03 V *vs.* NHE, respectively. According to the band gap (2.39, 1.59, 1.48 and 1.52 eV), the valence band (VB) positions of TiO_2_, PDI-1/TiO_2_, PDI-2/TiO_2_, and PDI-3/TiO_2_ were 1.06 V, 0.52 V, 0.44 V, and 0.49 V *vs.* NHE, respectively. The CB/VB of TiO_2_, PDI-1/TiO_2_, PDI-2/TiO_2_, and PDI-3/TiO_2_ were evaluated to be −1.33/1.06 V, −1.11/0.49 V, −1.04/0.32 V, and −1.01/0.44 V. The VB position of PDI-1/TiO_2_ was deeper than that of PDI-2/TiO_2_ or PDI-3/TiO_2_, which means that the oxidation ability of holes of PDI-1/TiO_2_ is stronger. It was noted that the VB positions of PDI-1/TiO_2_, PDI-2/TiO_2_, or PDI-3/TiO_2_ was shallower than that of hydroxyl radical (2.27 eV),^29^ and so it is possible that the hydroxyl radical cannot be generated by PDI-1/TiO_2_, PDI-2/TiO_2_, or PDI-3/TiO_2_. The CB position of singlet oxygen (0.34 eV) was shallower than that of PDI-1/TiO_2_, PDI-2/TiO_2_, or PDI-3/TiO_2_. This result revealed that singlet oxygen may be the main active substance in PDI-1/TiO_2_PDI-2/TiO_2_, or PDI-3/TiO_2_ composite for photocatalytic degradation of MB.

**Fig. 10:**
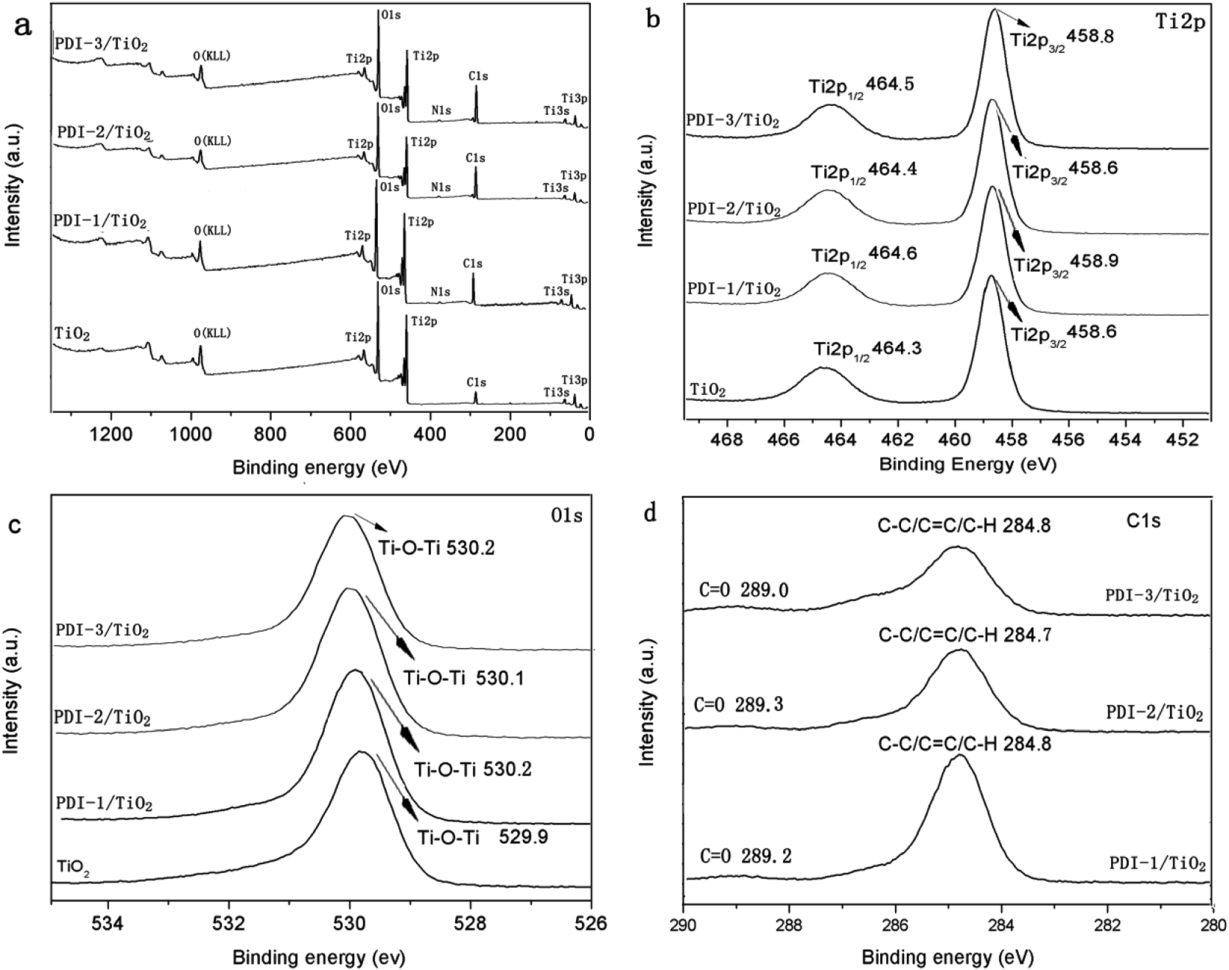
The XPS spectra of TiO_2_, PDI-1/TiO_2_, PDI-2/TiO_2_ and PDI-3/TiO_2_: (a) survey spectra, (b) Ti2p spectra, (c) O1s spectra and (d) C1s spectra.

To further illustrate the chemical state of these composites, X-ray photoelectron spectroscopy (XPS) analysis was carried out (Fig. 10). Fig. 10a showed that there were Ti, O, C, and N in these composites. Binding energy peaks of elements Ti, O, and C were shown in Fig. 10b–d, respectively. The Ti2p signal splits into two photoelectron peaks: Ti2p_1/2_ and Ti2p_3/2_, which appeared at the range of 464.3–464.6 eV and 458.6–458.9 eV, respectively.^50^ The O1s signal showed contributions at the range of 529.9–530.2 eV, which can be attributed to the O1s binding energy of oxygen in hydroxy group.^51^ The C1s spectra (Fig. 10d) showed two peaks with different values: 289.0–289.2 eV and 284.7–284.8 eV, which were identified as C=O and C–C/C=C/C–H functional groups, respectively.^52^ Compared with other composites, PDI-1/TiO_2_ composite had slightly larger binding energy peaks relative to that of pure TiO_2_. It indicated that there may be a strong interaction between self-assembled PDI-1 and TiO_2_, which could improve the charge separation efficiency in the system. The weak interaction between PDI-2 and TiO_2_ was due to the steric hindrance of the phenoxy substituent at bay position that prevented the perylene nucleus from contacting TiO_2_. Compared with PDI-1/TiO_2_, PDI-3/TiO_2_ had a larger Ti2p and O1s binding shift but its degradation efficiency was lower. This is because PDI-3 was amorphous, whereas PDI-1 had an ordered nanostructure. Conjugated PDI-1 can transfer electrons quickly from the semiconductor interior to the surface of TiO_2_. This property reduced the recombination of electrons and holes and improved the stability and photocatalytic activity of PDI-1/TiO_2_.

### 3.3. Mechanism of enhancement of photocatalytic activity

**Fig. 11:**
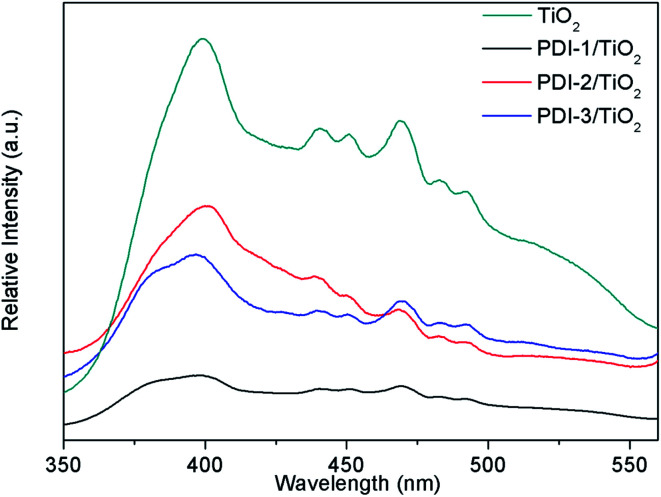
The photoluminescence spectra of TiO_2_, PDI-1/TiO_2_, PDI-2/TiO_2_ and PDI-3/TiO_2_ (*λ*_ex_ = 296 nm).

In order to understand the mechanism of the enhanced photocatalytic activity of PDI-1/TiO_2_, photoluminescence (PL) spectra of TiO_2_, PDI-1/TiO_2_, PDI-2/TiO_2_ and PDI-3/TiO_2_ were investigated. The PL spectra between 350–550 nm of TiO_2_, PDI-1/TiO_2_, PDI-2/TiO_2_, and PDI-3/TiO_2_ after excitation at 296 nm are shown in Fig. 11. TiO_2_ showed six major emission peaks, which were located at 399, 440, 451, 469, 483, and 492 nm, respectively. The peak at 399 nm was attributed to a direct transition from the conduction band to the valence band, while the remaining peaks are ascribed to exciton effects resulting from lattice defects and surface states. The PL peaks at 440, 451, and 469 nm were caused by inter-band transitions, and those at 483 and 492 nm were caused by intra-band transitions within the energy level traps or surface states.^53^ When PDI-1 and TiO_2_ were hybridized, the fluorescence emission showed a significant reduction. While when PDI-2 or PDI-3 was hybridized with TiO_2_, the emission intensity only changed slightly. The emission quantum yields (*Φ*) were 0.092%, 0.017%, 0.063%, 0.051% for TiO_2_, PDI-1/TiO_2_, PDI-2/TiO_2_, and PDI-3/TiO_2_, respectively. The results show that coupling of TiO_2_ and PDI-1 benefited electron–hole separation, which weakened the fluorescence intensity and improved the photocatalytic activity of PDI-1/TiO_2_.

**Fig. 12:**
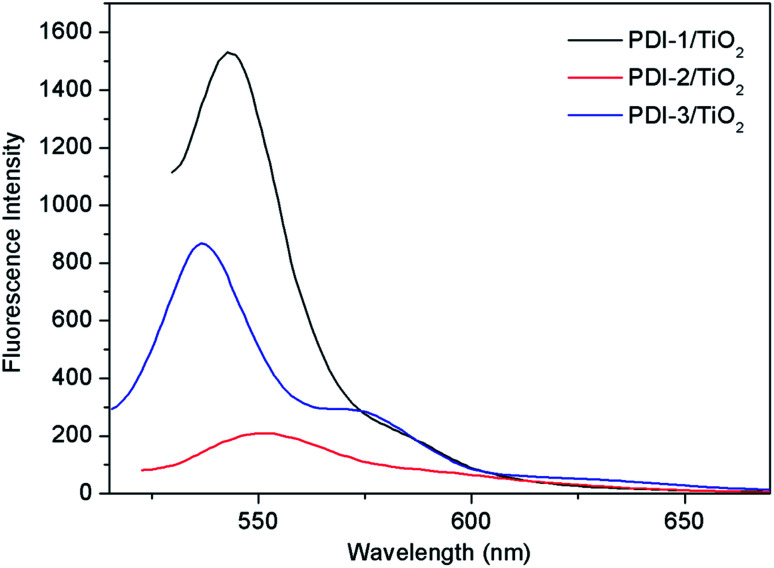
The photoluminescence spectra of PDI-1/TiO_2_, PDI-2/TiO_2_ and PDI-3/TiO_2_ (*λ*_ex_ = 493 nm).

The PL spectra of PDI-1/TiO_2_, PDI-2/TiO_2_, and PDI-3/TiO_2_ after excitation at 493 nm are shown in Fig. 12. The fluorescence intensity of PDIs depended on the density of transition carriers. On the other way, PDIs doping agents and aggregation were the direct factors to increase non-radiative transition, lead to energy loss, and inhibit radiative transition.^26^ It was observed that the emission of PDI-1/TiO_2_ had maximum intensity at 543 nm. The intensity of PDI-3/TiO_2_ hybrid was relatively lower, while it was higher than that of PDI-2/TiO_2_. The results indicated that PDI-1/TiO_2_ had the highest density of transition carriers. To interpret the photophysical properties in a more intuitive manner, the fluorescence lifetimes of PDI-1, PDI-2, PDI-3, PDI-1/TiO_2_, PDI-2/TiO_2_, and PDI-3/TiO_2_ were measured and shown in Fig. S13.† The PDIs and composites had single lifetime and decays were mono-exponential and the average fluorescence lifetimes were 3.41, 3.73, 3.52, 5.51, 5.30, and 5.43 ns, respectively. It was noted that the fluorescence lifetimes of PDI-1/TiO_2_ was 1.6 times longer than that of PDI-1. The addition of TiO_2_ would decrease electrons and holes recombination in the interfacial layer of PDI-1 shell due to ultrafast ET process.

**Fig. 13:**
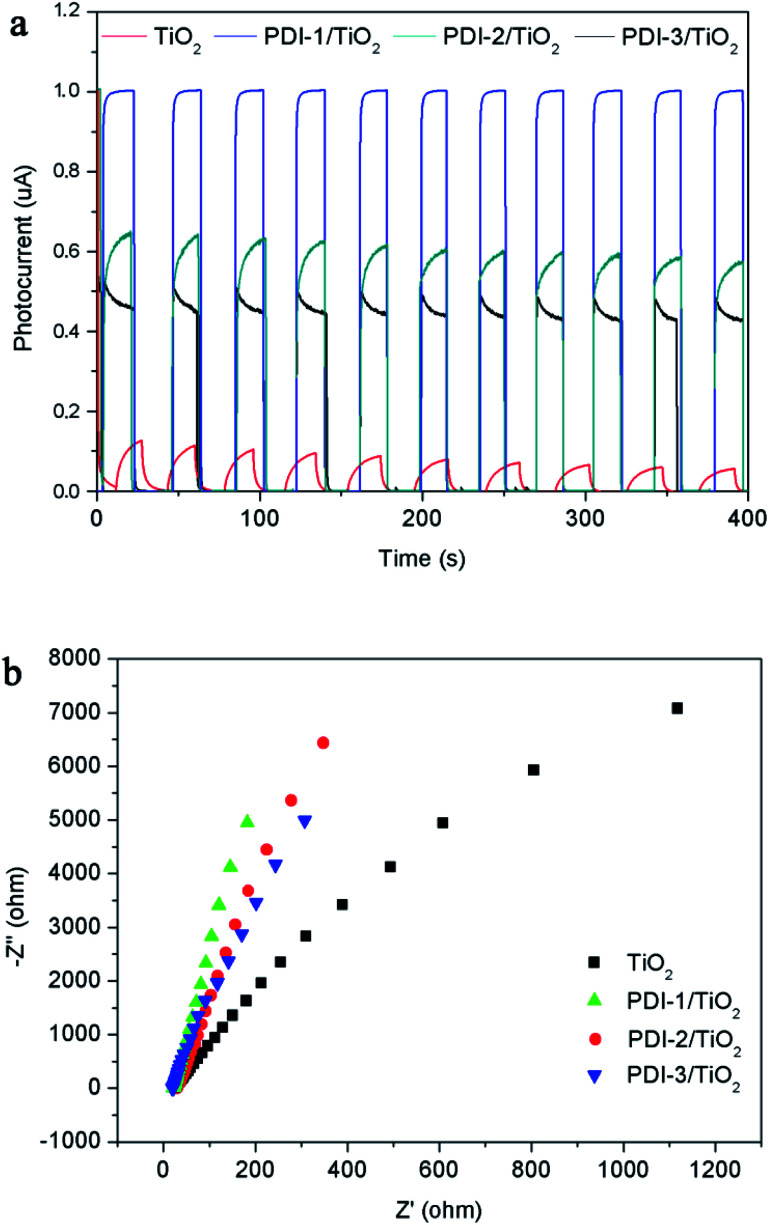
(a) Photocurrent response and (b) EIS spectra of TiO_2_, PDI-1/TiO_2_, PDI-2/TiO_2_ and PDI-3/TiO_2_.

The transient photocurrent responses of TiO_2_, PDI-1/TiO_2_, PDI-2/TiO_2_, and PDI-3/TiO_2_ and the corresponding electrochemical impedance (EIS) Nyquist plots are shown in Fig. 13. The photocurrent significantly reduced to 0 as soon as the light was turned off, and then it increased rapidly when the light was turned on again, which showing excellent stability and repeatability (Fig. 13a). The photocurrent of PDI-1/TiO_2_ was significantly higher than that of TiO_2_, PDI-2/TiO_2_, and PDI-3/TiO_2_, indicating that the interfacial electron transfer between photo-excited PDI-1 and TiO_2_ was more effective. Besides, Fig. 13b showed the EIS Nyquist plots of TiO_2_, PDI-1/TiO_2_, PDI-2/TiO_2_, and PDI-3/TiO_2_. The larger diameter of the semicircles represented higher impedance and slower interface charge recombination. The PDI-1/TiO_2_ had a larger diameter of the semicircle than TiO_2_, PDI-2/TiO_2_, or PDI-3/TiO_2_ in the middle frequency region, indicating that PDI-1/TiO_2_ had higher impedance and slower interface charge recombination, and thus improving its visible light photocatalytic activity.

The stability of PDI-1/TiO_2_ in MB degradation process has been tested under visible light through four consecutive cycles (Fig. S14†). After each run, the catalyst was centrifuged, washed with water, and dried in an oven at 100 °C for 1 h for recover. After four runs, 86.4% of the degradation activity still remained. Fig. S15† shows the FT-IR spectra and SEM images of the PDI-1/TiO_2_ photocatalyst before and after four recycling runs for the photocatalytic degradation of MB under solar irradiation. As can be seen from Fig. S15,† the FT-IR spectra and SEM images of the regenerated photocatalyst are almost the same as that of the fresh photocatalyst. These results indicate that PDI-1/TiO_2_ catalyst has high stability and is reusable.

Hybridization of PDI-1 with TiO_2_ improved the absorption and utilization of visible light. The degradation efficiencies of PDI-1/TiO_2_ and PDI-2/TiO_2_ catalysts were better than that of PDI-3/TiO_2_. PDI-1 nanorods and PDI-2 nanobelts can extend 1D charge carrier channel by π–π stacking and had large surface area. After hybridization of TiO_2_, the electron transferred to the TiO_2_ surface improved the photocatalytic activity of the composites. In this study, PDI-1 was loaded on the surface of TiO_2_ by hydrothermal synthesis method to produce an interaction. This made the contact between the two monomers more compacted and reduced the dissolution of PDI-1 single molecule. The oxygen on the perylene skeleton can also help firming up the stacking of the PDI-1 with TiO_2_ through extended π–π interaction and the electrostatic attraction between electron-rich atom O and electron-poor dye MB. Because of the band gap differences between PDI-1 and TiO_2_ and the unique structure of PDI-1/TiO_2_, it can absorb MB effectively, transfer charge rapidly, and increase the separation of electrons and holes dramatically. So, the photocatalytic activity of PDI-1/TiO_2_ could be greatly increased. Compared with PDI-1/TiO_2_, PDI-2/TiO_2_ showed high decolorization rate but low degradation efficiency. This is because the steric hindrance of phenoxy substituent at the bay position could prevent the perylene nucleus from contacting TiO_2_, making the interaction between PDI-2 and TiO_2_ weak. So, although the oxygen at the perylene core could absorb MB, PDI-2/TiO_2_ catalyst cannot degrade it effectively.

The PDI-1 nanostructure had an appropriate thickness so that the electrons excited in the interface layer can be injected into the conduction band of TiO_2_ to prevent electron–hole recombination. However, if the number of aggregates is excessively large to result in increased thickness and a stack that overlays some of the active sites, the photocatalytic activity will be reduced. Thus, there is a balance between charge separation and light absorption, and 0.01 wt% PDI-1/TiO_2_ might represent the balance point.

The UV-vis spectra of MB (10 mg L^−1^) solution with PDI-1/TiO_2_ catalyst at different visible irradiation times are shown in Fig. S16.† The spectra shape showed no significant change during irradiation, and the absorption peaks intensity at 291 nm and 664 nm decreased gradually and disappeared eventually. This indicates that the intermediate was not absorbed at the analytical wavelengths and MB was completely degraded.

**Fig. 14:**
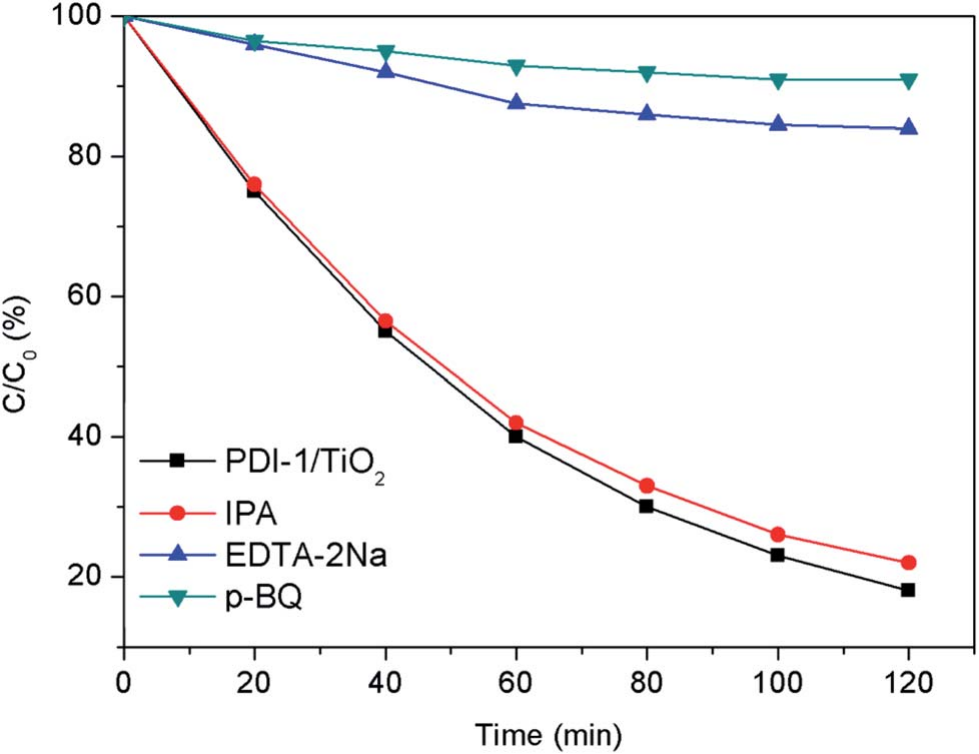
Plots of photo-generated active species trapped in the system of photodegradation of MB by PDI-1/TiO_2_ under visible light.

In order to study the photocatalytic mechanism of PDI-1/TiO_2_ photocatalyst, we used free radical capture experiment to identify active substances in photocatalytic process. Control experiments were carried out using *p*-benzoquinone (*p*-BQ) for quenching ˙O_2_^−^, disodium ethylenediamine tetraacetate (EDTA-2Na) for holes, and isopropanol (IPA) for ˙OH, respectively.^54–56^ As shown in Fig. 14, when EDTA-2Na was added, the degradation efficiency (16%) of MB with PDI-1/TiO_2_ decreased dramatically, implying that h^+^ was one of the main active radical in the photocatalytic degradation process. However, the degradation activity of the catalyst did not decrease after the addition of IPA, indicating that ˙OH was not the main active substances. When *p*-BQ was added in the reaction system, the degradation rate decreased from 82% to 9%, showing that ˙O_2_^−^ was also one of the main active substances. These data indicated that the main active substances for photocatalytic degradation of MB by PDI-1/TiO_2_ composite were h^+^ and ˙O_2_^−^.

**Fig. 15:**
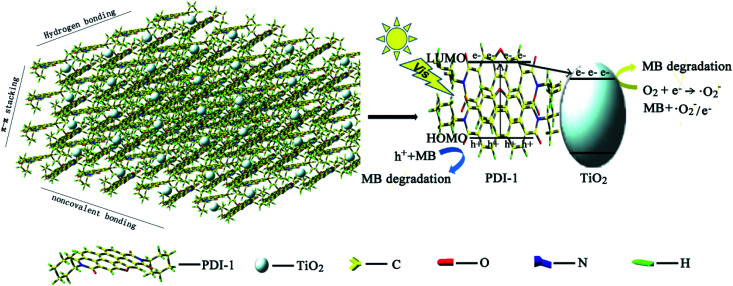
Photocatalytic degradation mechanism of PDI-1/TiO_2_ composite.

The HOMO/LUMO energy levels of PDI-1 obtained by DFT calculations at the B3LYP/6-31G* level are estimated to be −6.26/−3.62. Conduction band of TiO_2_ (rutile, −4.8 eV; anatase, −5.1 eV (ref. 57)) lies between the HOMO and LUMO levels of PDI-1. The HOMO/LUMO energy levels of PDI-2 and PDI-3 are estimated to be −6.52/−4.10 and −6.43/−3.89 eV, respectively. PDI-1 had higher HOMO and LUMO energy levels than PDI-2 and PDI-3, which would improve its hole-transfer ability. Molecular modeling shows that PDI-1 can reinforce the stacking of the molecules through noncovalent bonding, hydrogen bonding, and extended π–π stacking (Fig. 15). The crystal structure of PDI-1 exhibited a nearly planar molecular conformation composed of edge-to-face dimers. The dimer was herringbone filled structure with a plane spacing of 3.3 Å. The short planar distance between perylene chromophores facilitate charge carriers hopping from one molecule to the next. Under visible-light illumination, PDI-1 can be excited to generate electrons and holes. Since the LUMO potential of PDI-1 is more negative than the CB of TiO_2_, the photo-generated electrons of PDI-1 can be injected directly into the CB of TiO_2_ through the π–π conjugate structure. Some electrons transferred to TiO_2_ reacted with the pollutants, and some others reacted with O_2_ adsorbed on the catalyst surface to produce ˙O_2_^−^, which participated in photocatalytic reactions to oxidize pollutants into CO_2_ and H_2_O. At the same time, the holes could be easily moved to the surface and oxidize the adsorbed contaminants directly. This significantly increased the activity of the PDI-1/TiO_2_ photocatalyst for MB degradation under visible light.

In general, most of the photoelectrons and holes recombined quickly but only some were involved in photocatalytic degradation process. This led to a low quantum efficiency. When PDI-1 was combined with TiO_2_, PDI-1 aggregates acted as light-sensitizers and electrons would be transferred to the surface of TiO_2_ rapidly through the extended π–π structure in PDI-1. The extended π–π structure of PDI-1 facilitated it to gain good transmission holes performance and to transfer the photo-generated hole to the surface of the composite photocatalyst quickly. This reduced the photo-induced recombination rate of electrons and holes and further improved the photocatalytic activity of the catalyst.

## 4. Conclusions

Three novel visible-light-driven composite photocatalysts were reported. They were prepared by hydrothermal synthesis method, which made the contact between two monomers more compacted and reduced the dissolution of PDIs single molecule. A series of chemical characterizations and MB degradation experiments showed that PDI-1/TiO_2_ had excellent photocatalytic activity and stability. The extended π–π stacking of self-assembled PDI-1 and strong interactions between self-assembled PDI-1 and TiO_2_ play significant roles in accelerating charge transfer and decreasing the recombination of photogenerated electron–hole pairs. The tests of radical scavengers confirmed that h^+^ and ˙O_2_^−^ were the main active substances for the degradation of MB. The characterization of the doped pigments was achieved using X-ray powder diffraction, DRS spectroscopy, FT-IR spectrum, XRD spectra, and photoluminescence measurements. Valence state and size of the binding energy were analyzed using XPS spectra. This work provides a practical way to improve the performance of traditional organic and inorganic composite photocatalysts by designing nano-structured heterocyclic annulated PDIs doped TiO_2_ photocatalysts. Also, it provides an efficient way to obtain stable PDIs doped TiO_2_ photocatalyst by hydrothermal synthesis.

## Conflicts of interest

There are no conflicts to declare.

## Supplementary Material

RA-010-D0RA03421E-s001
