# Supplementary material for: Real roles of perylene diimides for improving photocatalytic activity
Source: RSC Adv. 2020 Jun 17;10(39):23024–37. doi: 10.1039/d0ra03421e (PMC9054670; doi:10.1039/d0ra03421e)
Supplement: RA-010-D0RA03421E-s001 [file RA-010-D0RA03421E-s001.pdf]

Electronic Supplementary Material (ESI) for RSC Advances.
This journal is © The Royal Society of Chemistry 2020

Supporting Information

Real roles of perylene diimides for improving photocatalytic activity

Fengxia Zhang*, Wenjing Li, Tianyi Jiang, Xuemei Li, Yuanyuan Shao, Yongshan Ma*, Junsen Wu*

School of Municipal and Environmental Engineering, Shandong Jianzhu University, Jinan 250101, Shandong, China.

* Corresponding author. E-mail addresses: zhangfengxia19@sdjzu.edu.cn (F. Zhang), mlosh@sdjzu.edu.cn (Y. Ma), wujunsen@sdjzu.edu.cn (J. Wu).

Experimental section

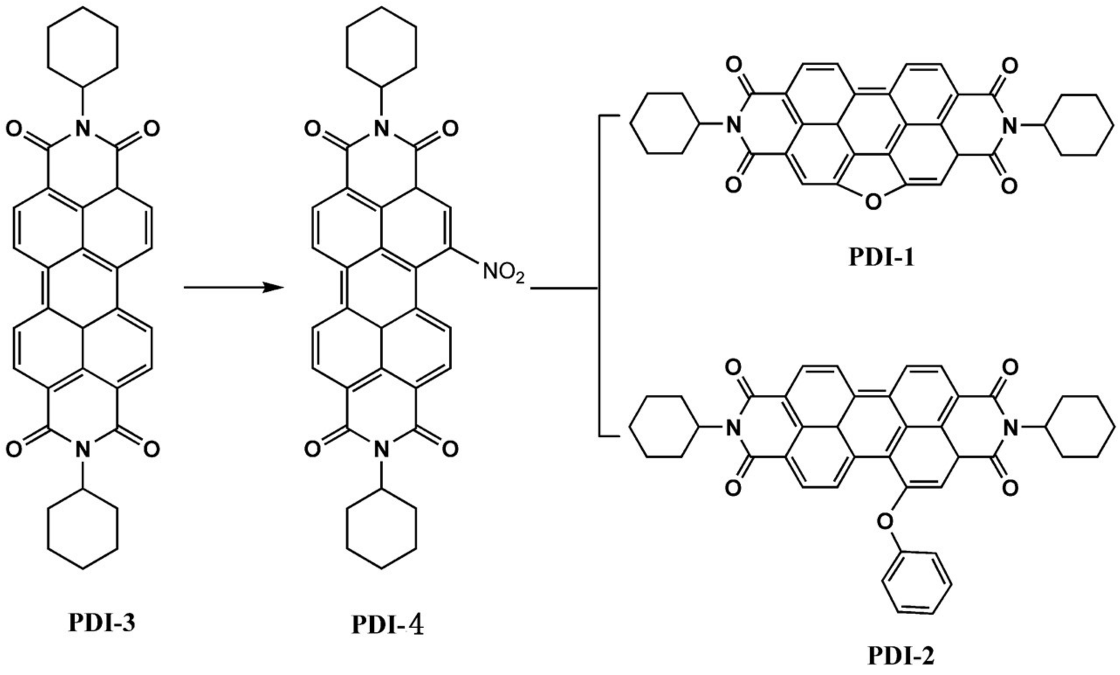

Fig.S1 Synthesis routes of PDI-1 and PDI-2.

General procedure for the synthesis of PDI-1

PDI-4 was achieved by a reaction of PDI-3 with cerium (IV) ammonium nitrate ($Ce(NH_4)_2(NO_3)_6$) and 96% nitric acid under ambient temperature in dichloromethane with a high yield (> 90%). PDI-4 (120mg, 0.2mmol) was dissolved in N-methylpyrrolidone (NMP, 10ml). The resulting solution was stirred at 180 ℃ for 5h until the starting material could not be detected by TLC, and then poured into 100ml of 2 M HCl. The precipitate was collected by vacuum filtration and washed with water. After solvent was removed under vacuum, the crude product was purified by silica gel column chromatography with eluent dichloromethane /petroleum ether (5/2) to afford a bright yellow solid of 34 mg (30%) was obtained. ^{1}H-NMR ($CHCl_3$, TMS, ppm): δ = 8.95 (d, 2H), 8.83 (d, 4H), 5.16 (m, 2H), 2.66-2.63 (m, 4H), 1.94-1.55(m, 8H), 1.41-1.26(m, 8H). ^{13}C NMR (75 MHz, $CDCl_3$, ppm): δ = 164.78, 163.74, 154.15, 133.93, 130.38, 126.46, 125.03, 124.36, 119.34, 54.50, 29.27, 26.65, 25.48. FT-IR (KBr, cm^{-1}): ν = 2922, 1689, 1649, 1611, 1415, 1341, 1307, 1259, 1179, 1000, 896, 852, 805, 735, 628, 576, 449, 416. MS (MALDI-TOF): m/z 569.2 $[M+H]^+$.

General procedure for the synthesis of PDI-2.

PDI-4 (120 mg, 0.2 mmol), phenol (95 mg, 1.0 mmol), 200 mg K_2CO_3 and the catalyzed KI were suspended in 10 mL anhydrous N-methylpyrrolidone (NMP). The resulting mixture was stirred at 25 ℃ for 6 h under argon atmosphere, and then poured into MeOH (7 mL) and 2 M HCl solution (30 mL). The precipitate was collected by vacuum filtration, washed with methanol, and then dried in vacuum. The crude product was further purified by silica gel column chromatography with eluent dichloromethane /petroleum ether (4:1 by volume). After solvent was removed, a red solid of 116 mg (90%) was obtained. ^{1}H-NMR (300 MHz, $CHCl_3$, TMS, ppm): δ = 9.44 (d, J = 6.0 Hz, 1H), 8.62-8.51 (m, 5H), 8.19 (s, 1H), 7.45 (m, 2H), 7.30 (m, 1H), 7.15 (m, 2H), 5.01 (m, 2H), 2.53-2.36 (m, 4H), 1.91-1.75 (m, 8H), 1.43-1.25 (m, 8H). FT-IR (KBr, cm^{-1}): ν = 2931, 2849, 1696, 1649, 1601, 1482, 1402, 1338, 1251, 1187, 813, 757. MS (MALDI-TOF): m/z 647.2 $[M+H]^+$.

Samples Characteriaztions Profiles

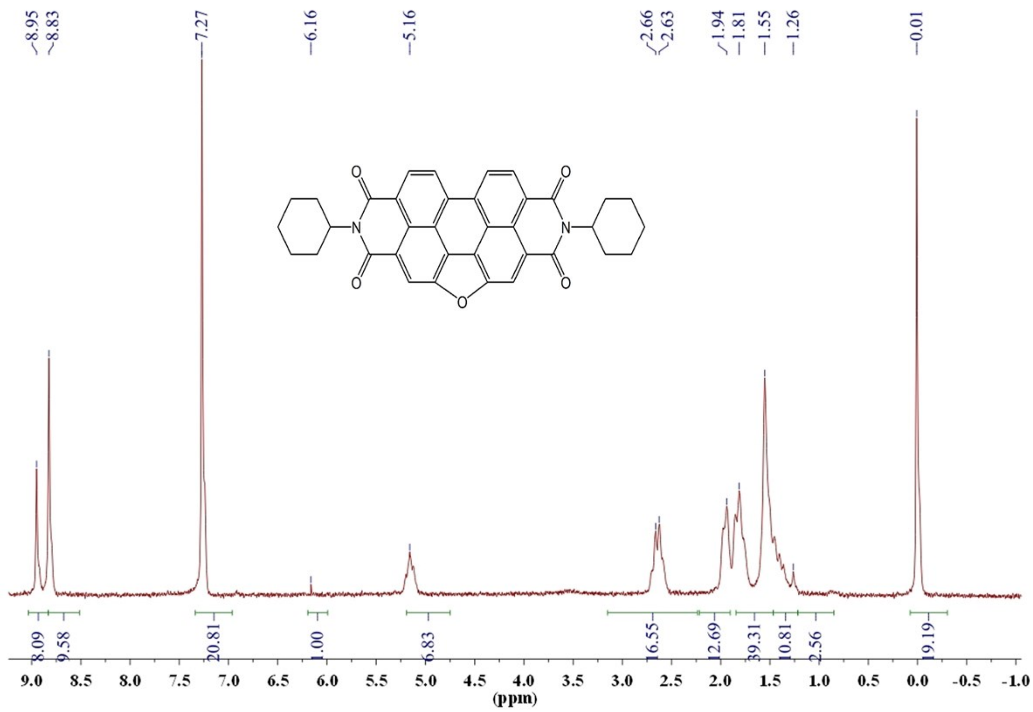

Figure S2. ^{1}H NMR spectrum of PDI-1.

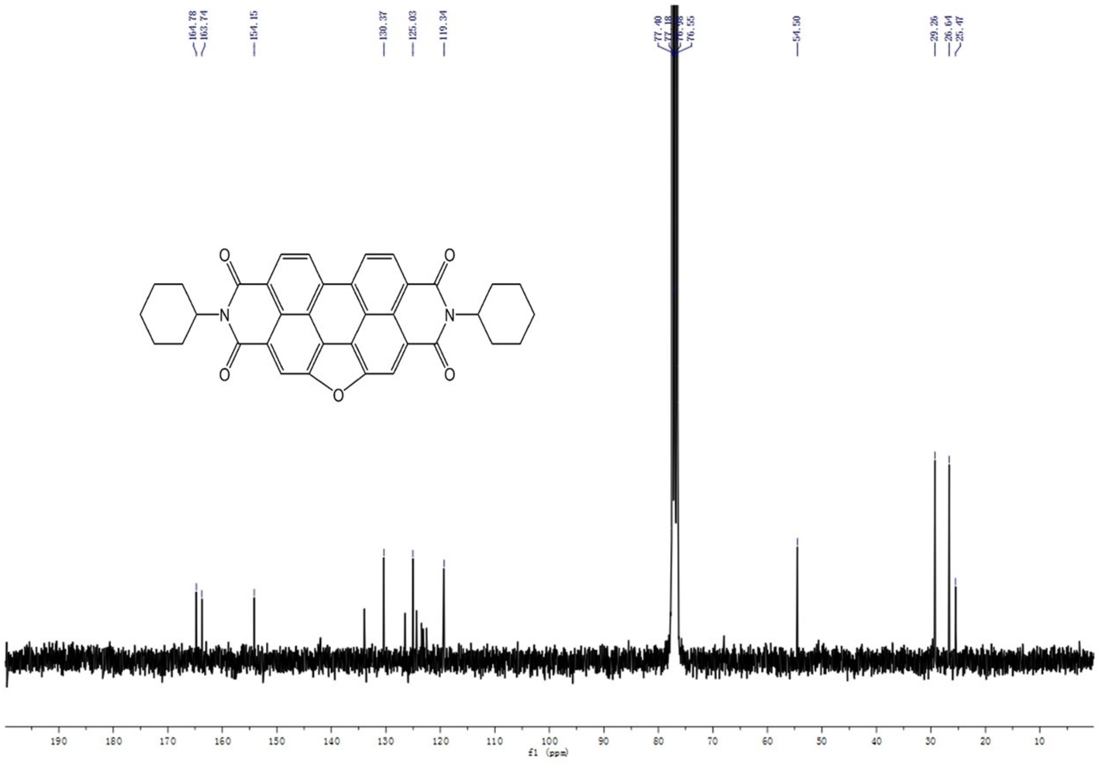

Figure S3. ^{13}C NMR spectrum of PDI-1.

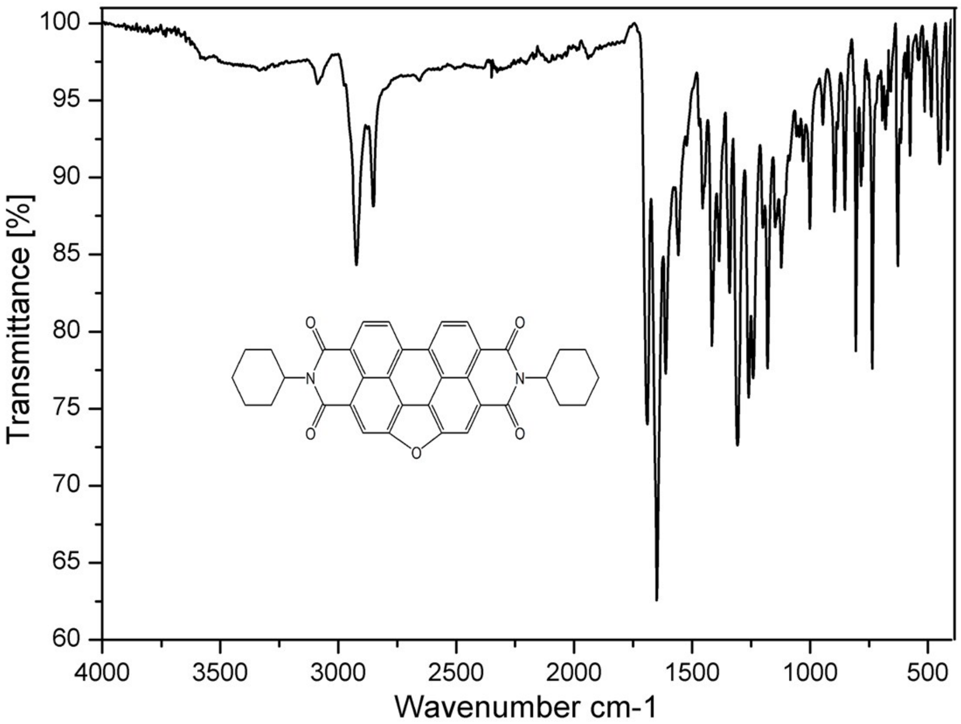

Figure S4. FT-IR profile of PDI-1.

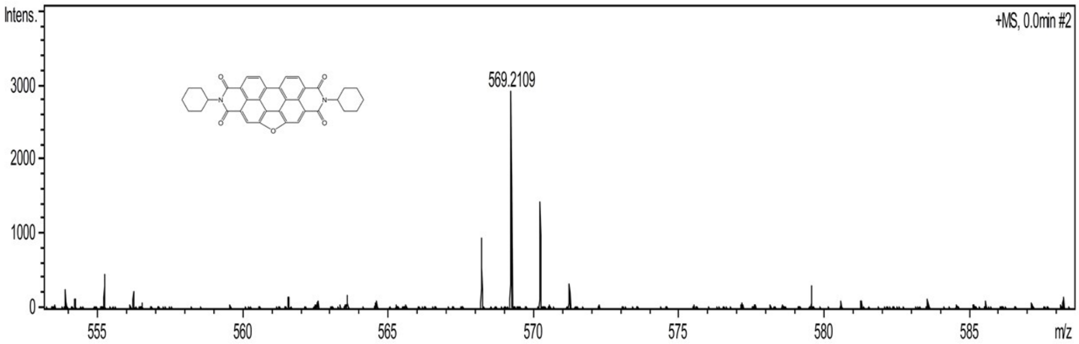

Figure S5. HRMS profile of PDI-1.

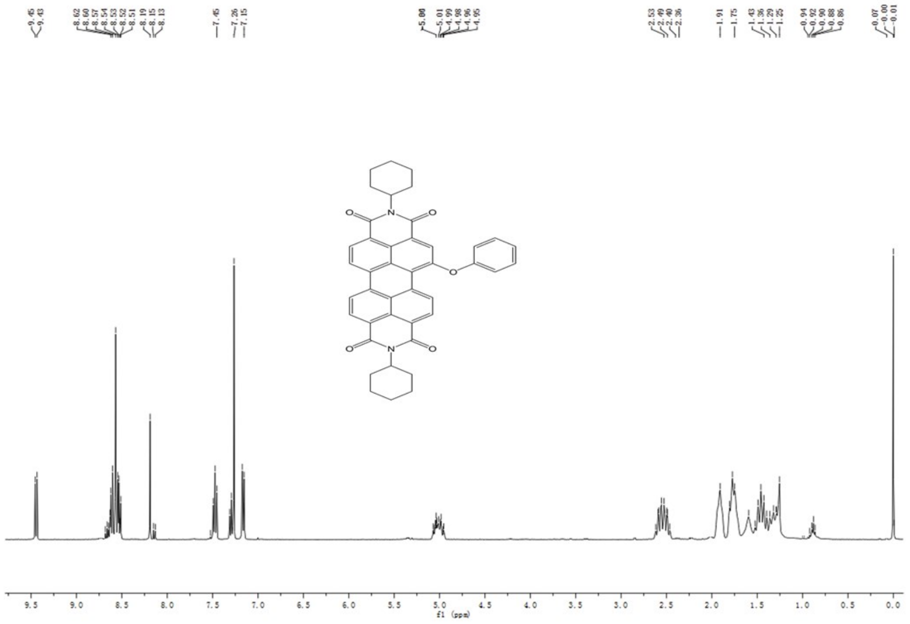

Figure S6. ^{1}H NMR spectrum of PDI-2.

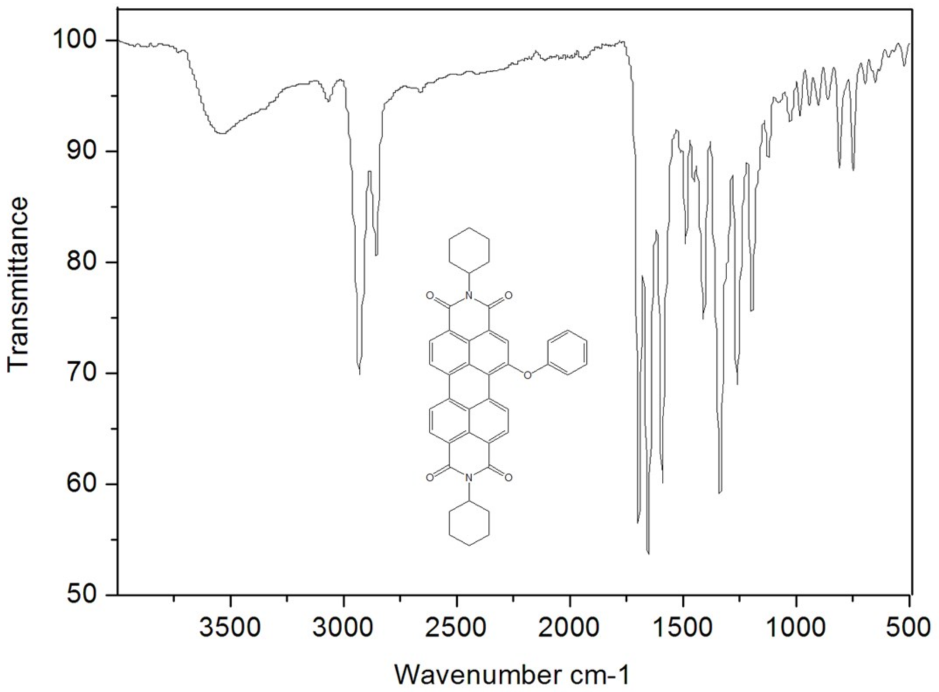

Figure S7. FT-IR profile of PDI-2.

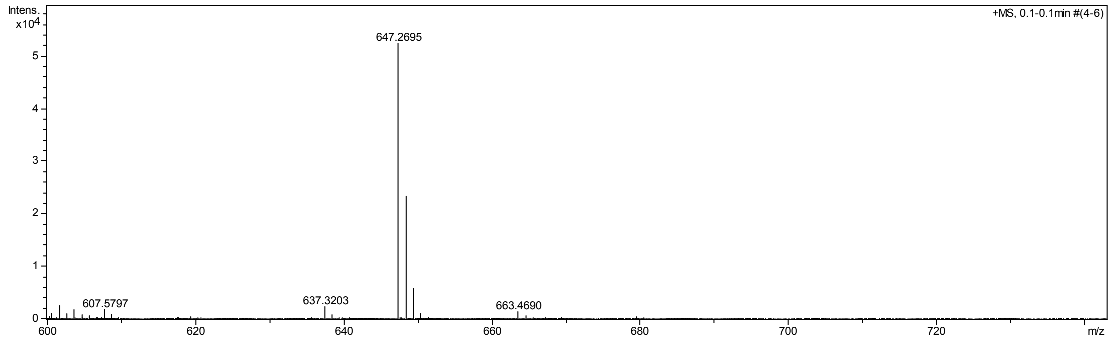

Figure S8. HRMS profile of PDI-2.

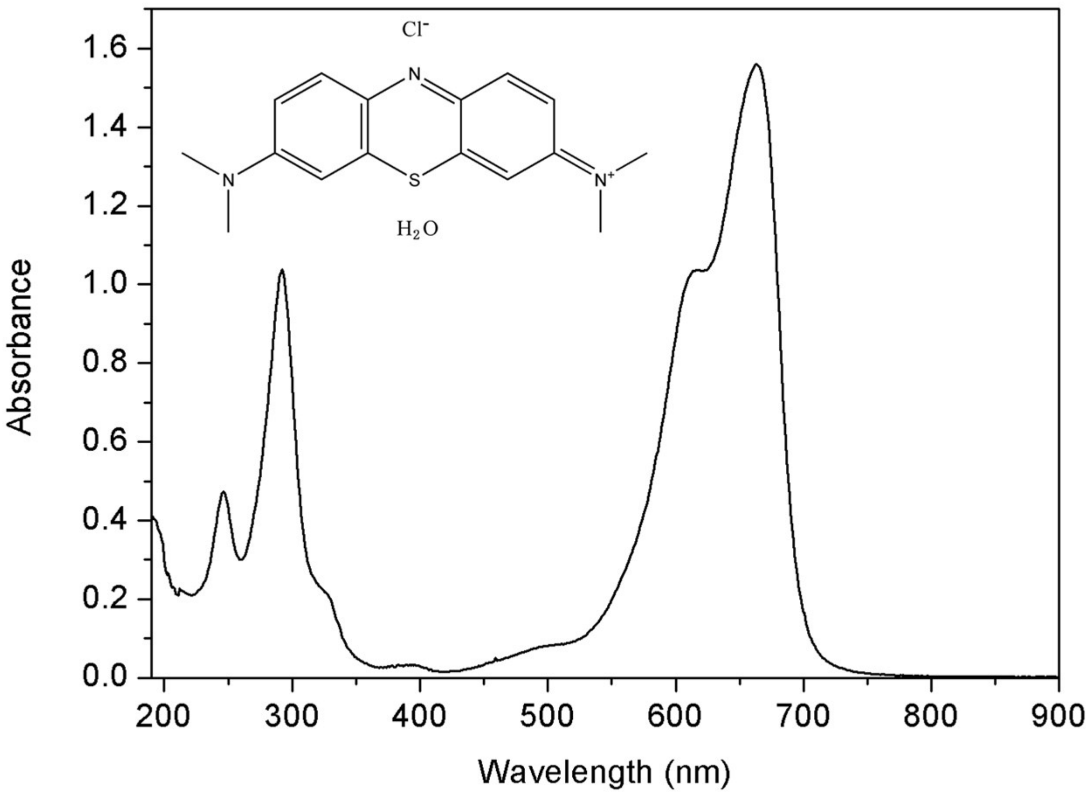

Figure S9. Dye MB structure and UV-visible absorption spectrum.

Table S1 Degradation rate constants of different photocatalysts.

photocatalysts	k min^{-1}
0.005 wt% PDI-1/TiO_2	0.0186
0.01 wt% PDI-1/TiO_2	0.0287
0.02 wt% PDI-1/TiO_2	0.0286
PDI-2/TiO_2	0.0216
PDI-3/TiO_2	0.0199

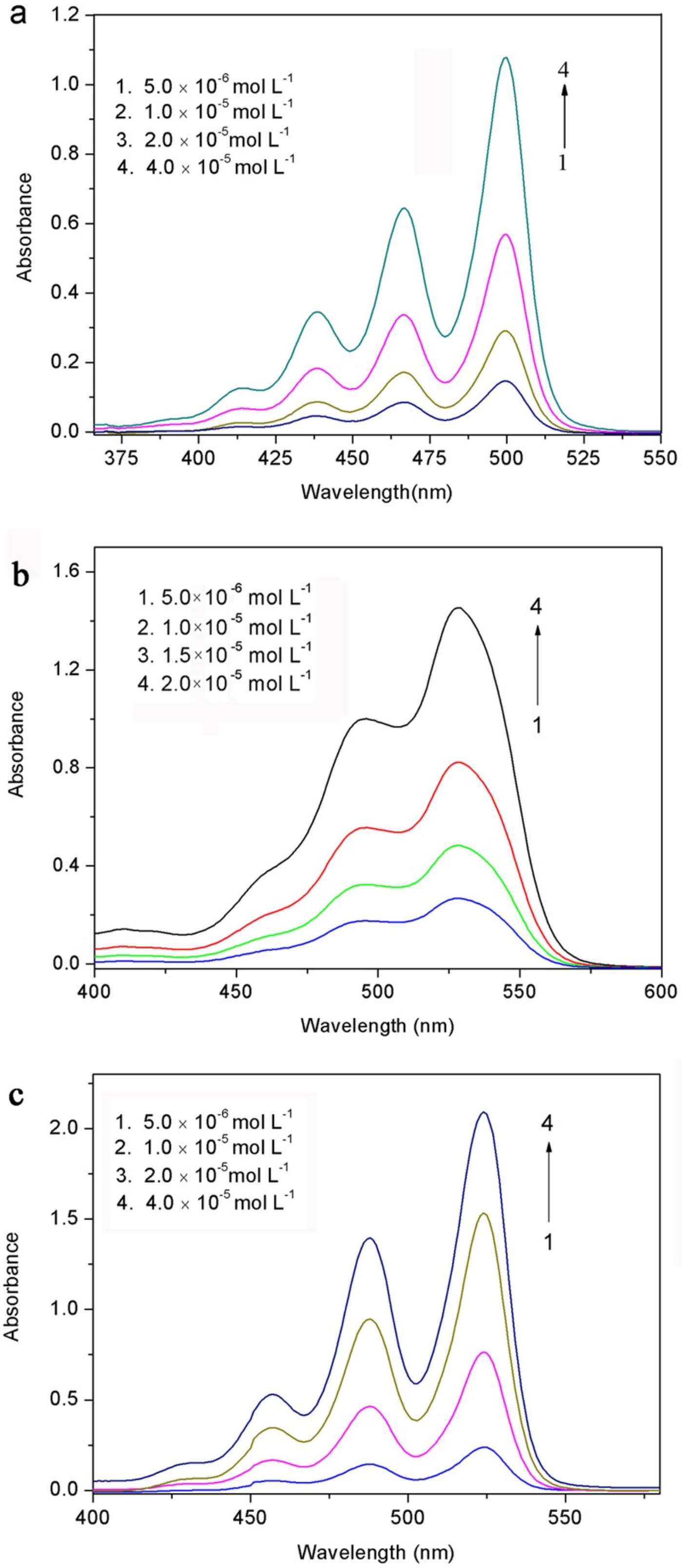

Figure S10 Absorption spectra of PDI-1 (a), PDI-2 (b) and PDI-3 (c) in dichloromethane at various concentrations.

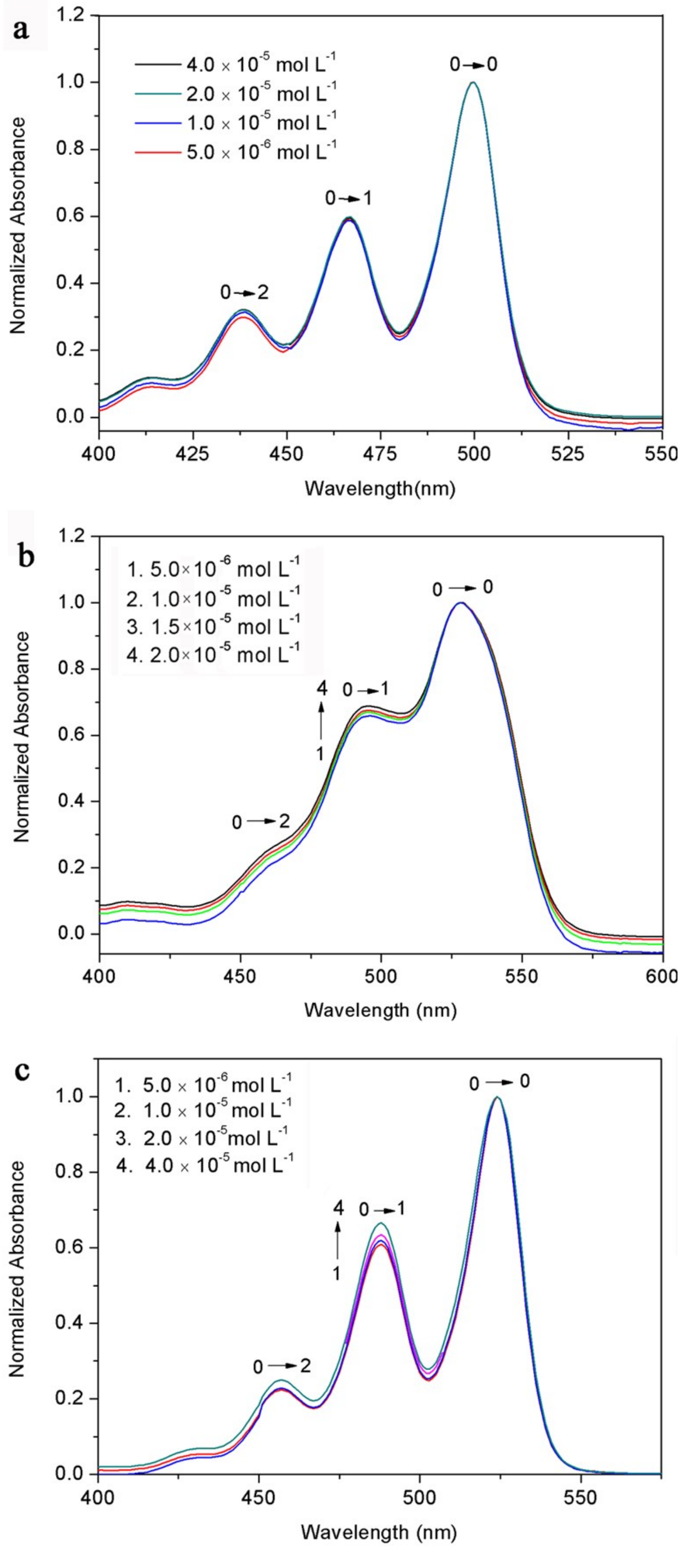

Figure S11 Normalized absorption spectra of PDI-1 (a), PDI-2 (b) and PDI-3 (c) in dichloromethane at various concentrations.

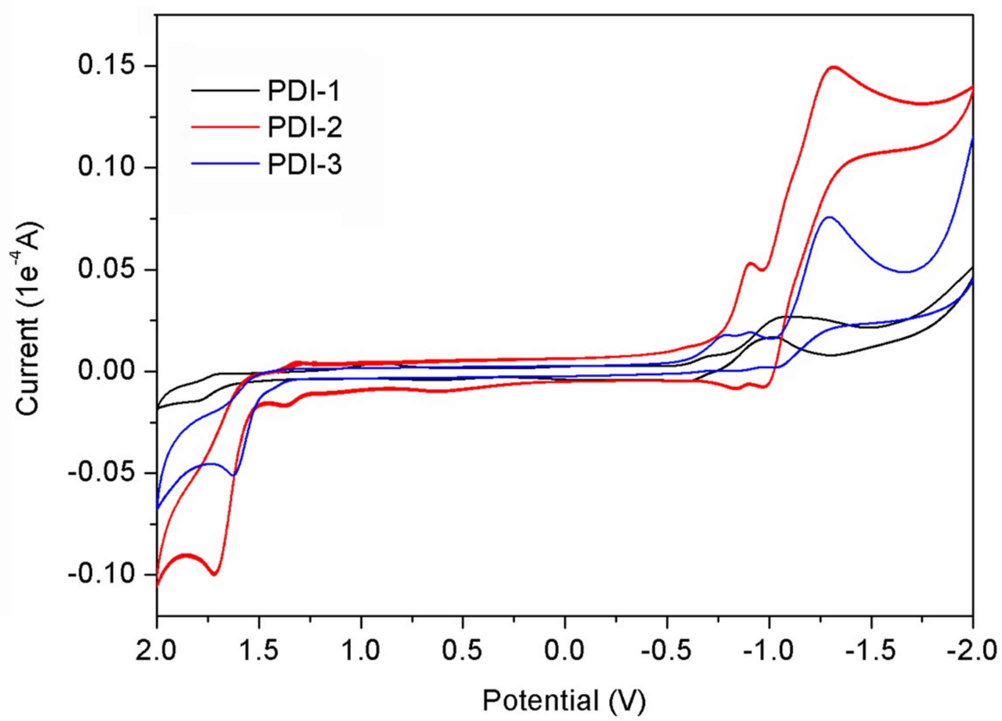

Figure S12. Cyclic voltammogram of PDI-1, PDI-2 and PDI-3 in dichloromethane.

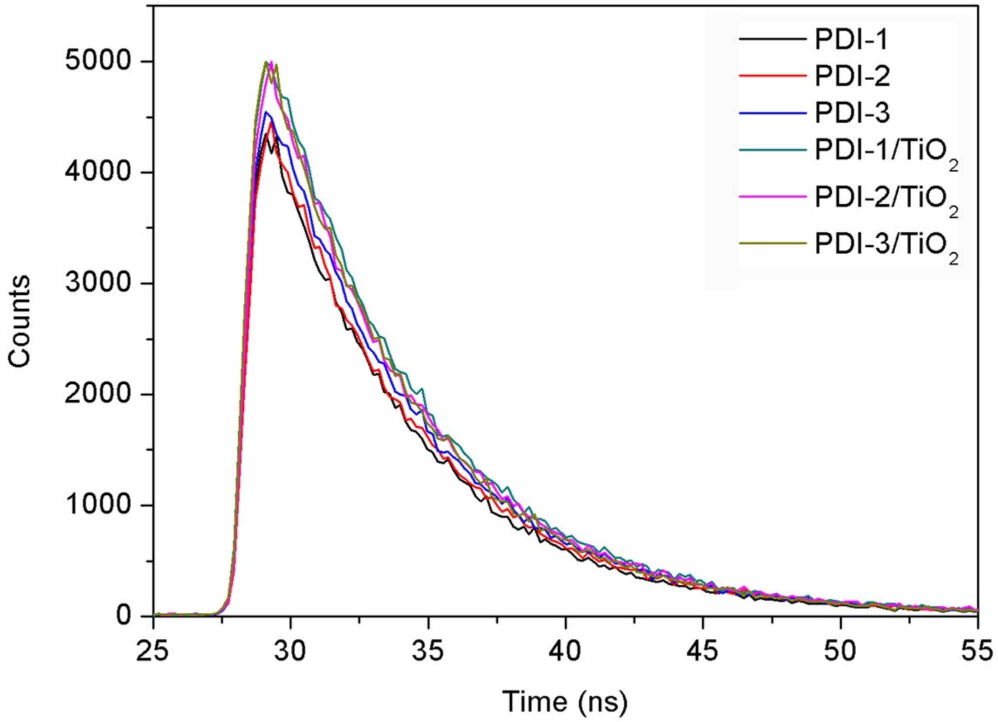

Figure S13. Fluorescence decay transients measured at 493 nm PDI-1, PDI-2, PDI-3, PDI-1/TiO_2, PDI-2/TiO_2, and PDI-3/TiO_2.

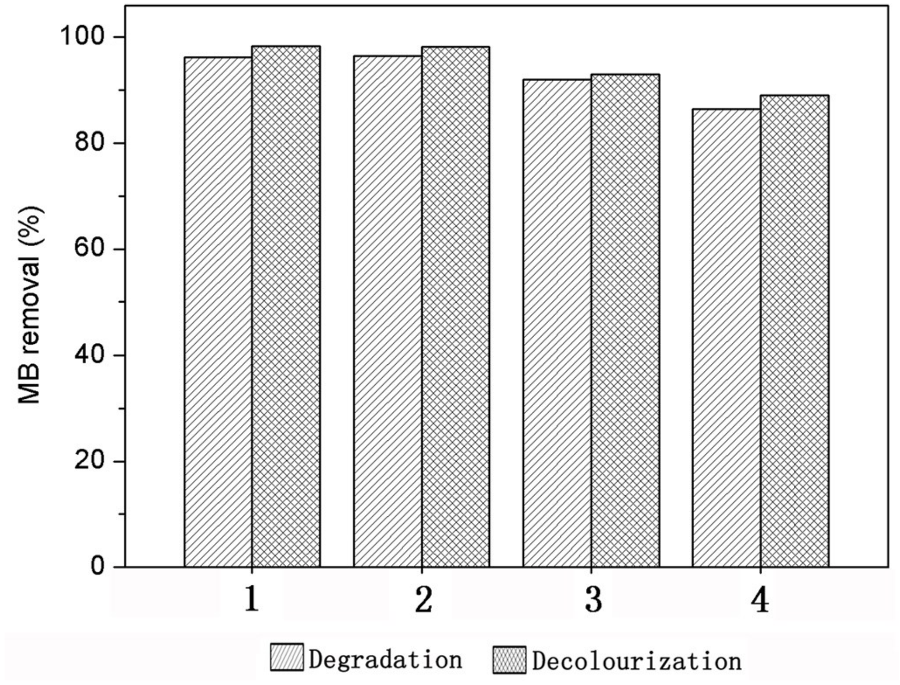

Figure S14. Catalyst reusability with PDI-1/TiO_2 catalysts under visible light; [MB] = 10 mg/L, pH = 7, catalyst suspended = 1g/L, irradiation time = 120 min.

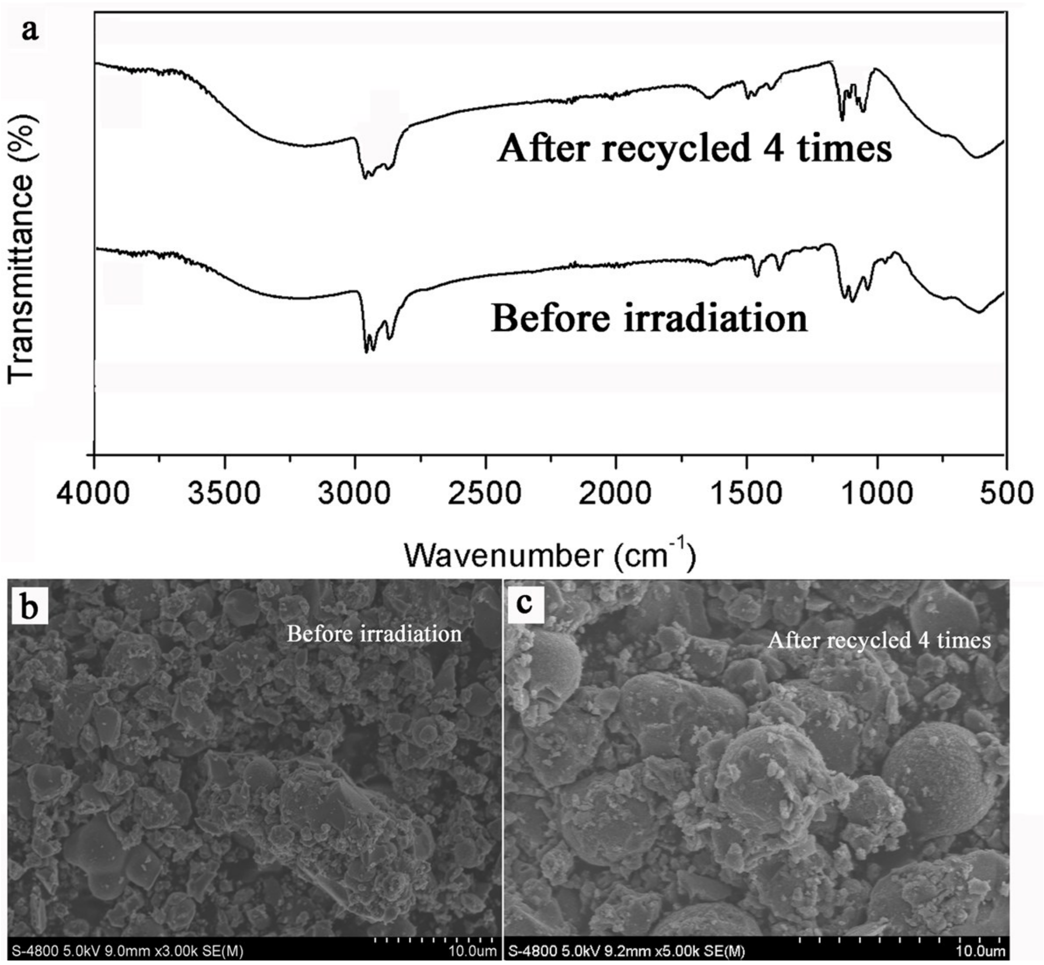

Figure S15. FT-IR spectra (a) and SEM images (b, c) of PDI-1/TiO_2 photocatalyst before and after 4 cyclicruns.

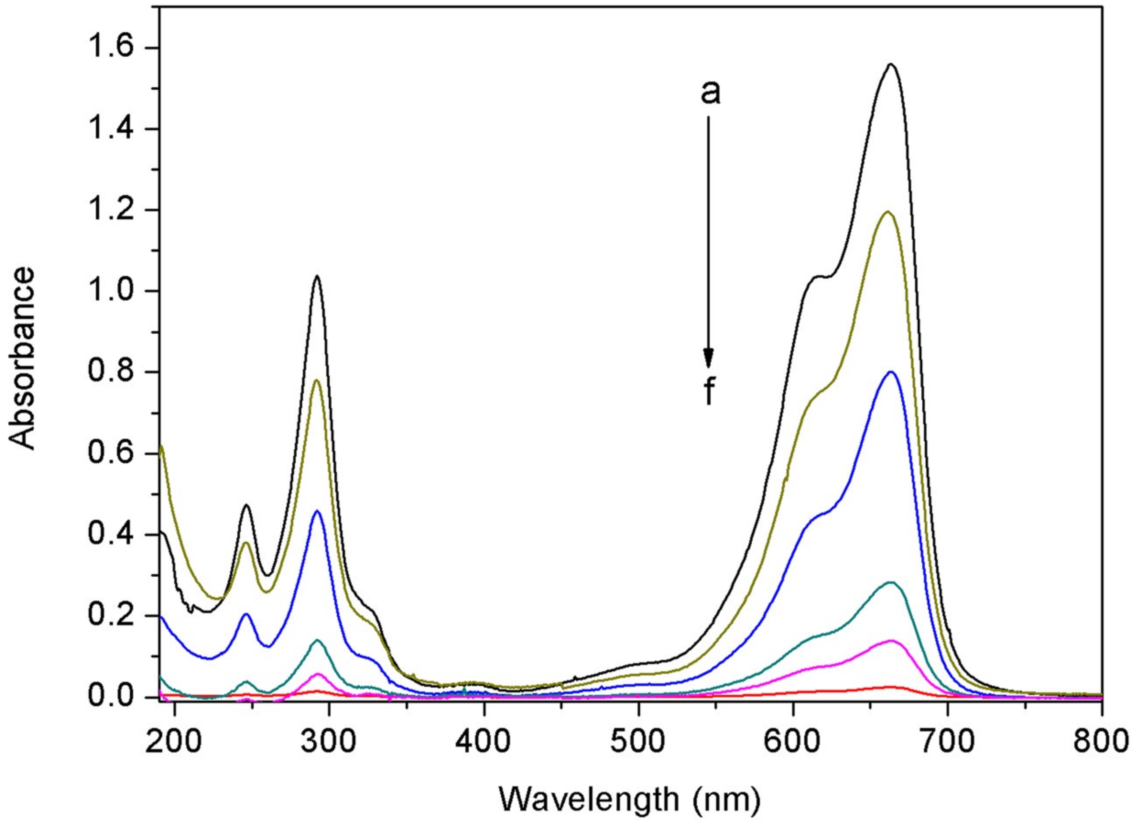

Figure S16. The changes in UV-vis spectra of MB on irradiation with visible light in the presence of PDI-1/TiO2. a) 0 min, b) 20 min, c) 40 min, d) 60 min, e) 80 min and f) 120 min. [MB] = 10 mg/L, pH = 7, catalyst suspended = 1g/L.
